# A Homozygous Deep Intronic Variant Causes Von Willebrand Factor Deficiency and Lack of Endothelial-Specific Secretory Organelles, Weibel–Palade Bodies

**DOI:** 10.3390/ijms23063095

**Published:** 2022-03-13

**Authors:** Hamideh Yadegari, Muhammad Ahmer Jamil, Natascha Marquardt, Johannes Oldenburg

**Affiliations:** Institute of Experimental Haematology and Transfusion Medicine, University Hospital Bonn, 53127 Bonn, Germany; muhammad.jamil@ukbonn.de (M.A.J.); natascha.marquardt@ukbonn.de (N.M.)

**Keywords:** von Willebrand factor, von Willebrand disease, deep intronic mutation, next-generation sequencing, Weibel–Palade bodies, angiopoietin-2, ECFCs

## Abstract

A type 3 von Willebrand disease (VWD) index patient (IP) remains mutation-negative after completion of the conventional diagnostic analysis, including multiplex ligation-dependent probe amplification and sequencing of the promoter, exons, and flanking intronic regions of the VWF gene (*VWF*). In this study, we intended to elucidate causative mutation through next-generation sequencing (NGS) of the whole *VWF* (including complete intronic region), mRNA analysis, and study of the patient-derived endothelial colony-forming cells (ECFCs). The NGS revealed a variant in the intronic region of *VWF* (997 + 118 T > G in intron 8), for the first time. The bioinformatics assessments (e.g., SpliceAl) predicted this variant creates a new donor splice site (ss), which could outcompete the consensus 5′ donor ss at exon/intron 8. This would lead to an aberrant mRNA that contains a premature stop codon, targeting it to nonsense-mediated mRNA decay. The subsequent quantitative real-time PCR confirmed the virtual absence of VWF mRNA in IP ECFCs. Additionally, the IP ECFCs demonstrated a considerable reduction in VWF secretion (~6% of healthy donors), and they were devoid of endothelial-specific secretory organelles, Weibel–Palade bodies. Our findings underline the potential of NGS in conjunction with RNA analysis and patient-derived cell studies for genetic diagnosis of mutation-negative type 3 VWD patients.

## 1. Introduction

Von Willebrand factor (VWF), a large multimeric plasma glycoprotein, plays essential roles in hemostasis by mediating platelet aggregation and serving as a carrier for coagulation factor VIII (FVIII) [1,2]. Deficient VWF leads to von Willebrand disease (VWD), the most recurrent inherited bleeding disorder, which is mainly characterized by mucocutaneous bleeding and bleeding from an injury or after a surgery [3,4]. The VWD is classified as quantitative types 1 and 3 (reduction or virtual absence of VWF, respectively), as well as qualitative type 2 (defects in VWF binding functions). Type 3 VWD, the most severe form of the disease, is caused by very low or no circulating VWF [5,6,7]. The type 3 VWD is inherited as an autosomal recessive trait caused by homozygous or compound heterozygous VWF gene (*VWF*) mutations, leading to null alleles [8].

The *VWF* comprises 52 exons, and it is transcribed into an 8.8 kb mRNA, encoding a precursor VWF [9]. The VWF precursor contains a signal peptide, a propeptide (D1-D2 domains), and a mature VWF (domains of D′-D3-A1-A2-A3-D4-C1-C2-C3-C4-C5-C6-CK) [10]. VWF is produced in endothelial cells (ECs) and megakaryocytes. In the ECs, VWF is stored in EC-specific secretory organelle, Weibel–Palade bodies (WPBs). The primary product of VWF undergoes modifications including dimerization (through CK domains), multimerization (by disulfide bonds between D3 domains), and proteolytic cleavage of the propeptide, followed by tubular packing of multimers and targeting into WPBs [11,12,13,14,15]. WPBs are elongated storage/secretory organelles that contain VWF, a prerequisite for WPB biogenesis, as well as inflammatory and proangiogenic molecules such as P-selectin and angiopoietin-2 (Ang2) [16,17,18,19]. 

Gene analysis of several type 3 VWD cohorts of various ethnic populations previously detected mutations (homozygous or compound heterozygous variants) in more than 85% of cases. In the remaining patients, either no causative variation was detected or only one *VWF* mutation was identified [20,21,22,23,24,25,26]. In our cohort of type 3 VWD patients, the mutation detection rate was about 94%, following completing genetic diagnostic tests including multiplex ligation-dependent probe amplification (MLPA) as well as Sanger DNA sequencing of the promotor, coding regions, and conserved consensus splice site (ss) (exon/intron borders) of *VWF* [20,27]. It is proposed that the causative mutations in type 3 mutation-negative cases or heterozygous cases might be located within deep intronic regions or distant regulatory sequences [28]. Nevertheless, to date, there is no report of confirmation of any gene variant outside of the *VWF* coding region or its consensus ss in type 3 VWD patients. In this study, we aimed to elucidate disease genetics etiology in one type 3 VWD index patient (IP), who was found to be without any causative mutations by routine genetic analysis, through screening of the *VWF* deep intronic region by next-generation sequencing (NGS) and an in-depth study of the patient-derived endothelial colony-forming cells (ECFCs), which are true-to-nature cell models [29].

## 2. Results

### 2.1. Patient Characterization

#### 2.1.1. Patient Phenotype Description

The 26-year-old female IP is diagnosed to have type 3 VWD, due to no detectable VWF in plasma (VWF antigen (VWF:Ag) level < 4%). The IP was referred to the Hemophilia Centre at University Hospital Bonn at an early age (one year old), due to bleeding complications (Table 1). The IP has a history of frequent epistaxis, easy bruising, bleeding following injury, and menorrhagia. The last, heavy and prolonged bleeding during the menstrual period, was the most prominent bleeding manifestation in the IP, which led to iron-deficient anemia. Although a life-long prophylaxis replacement therapy was recommended to be highly desirable, the IP refused because of discomfort associated with injections. Nevertheless, over some time, she underwent prophylaxis replacement therapy, receiving plasma-derived VWF/FVIII concentrates, and for some other times, she received on-demand antihemorrhagic treatments including antifibrinolytics (tranexamic acid), oral contraceptives, and replacement therapy.

#### 2.1.2. Patient Genotype: Detection of a Deep Intronic Variant, Creating New Donor Splice Site

**Figure 1:**
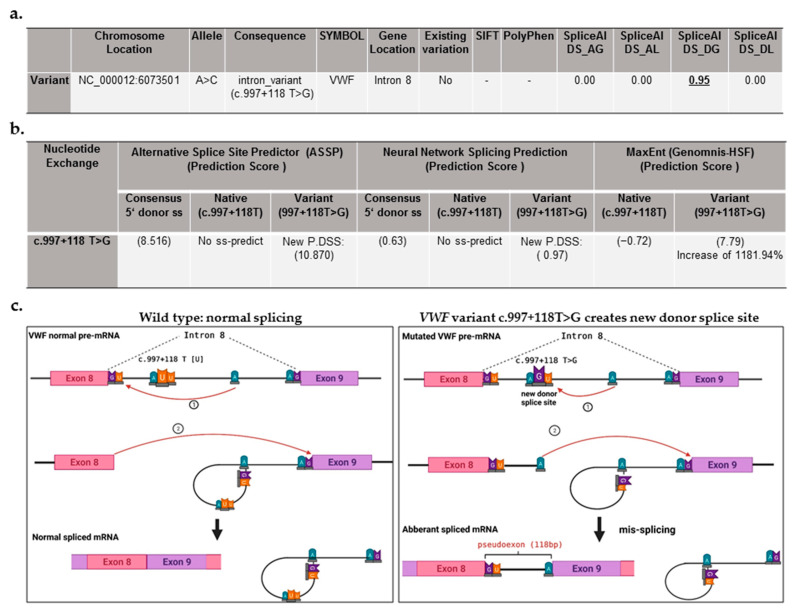
Detection of a deep intronic variant in von Willebrand factor (VWF) gene after executing next-generation sequencing (NGS) and subsequent bioinformatic analysis. (**a**) Data obtained from analysis of variant c.997 + 118 T > G, detected by NGS, using Ensemble Variant Effect Predictor (VEP), including variant annotation, variant location, the existing variant in public variant databases, and predicting its pathogenic effect. Genomic location of VWF variant is annotated based on latest assembly (GRCh38/hg38: chr12: 5,948,877–6,124,770). No: not existing in the public variant databases. SpliceAl (Illumina artificial intelligence splicing prediction software) prediction scores include DS_AG (delta score for acceptor gain), DS_AL (delta score for acceptor loss), DS_DG (delta score for donor gain), and DS_DL (delta score for donor loss). The calculated scores range from 0 to 1 and can be interpreted as the probability of the variant being splice-altering. The suggested cutoffs are 0.2 (high recall), 0.5 (recommended), and 0.8 (high precision). The VEP-SpliceAl tool predicted the variant c.997 + 118 T > G induces the gain of a donor splice site, providing the DS_DG score of 0.95. -, not applicable, the SIFT and Polyphen predict the effect of exonic variants on protein function/structure. (**b**) Summary of bioinformatic analysis (by Neural Network Splicing, Alternative Splice Site Predictor, and plug-in MaxEnt of the Human Splicing Finder (HSF)), predicting the impact of the VWF gene (*VWF)* variant c.997 + 118 T > G on splicing processing. ss, splicing site; P.DSS, potential donor splice site. (**c**) Schematic image of normal splicing of the wild-type VWF mRNA at exon/intron 8 junction (left side) as well as the mis-splicing due to the variant c.997 + 118 T > G, embedded in intron 8 of *VWF*, which creates a new donor splice site, leading to an aberrant mRNA with exon elongation (a pseudoexon of 118 base pairs (bp)).

No causative variation was identified after the routine genetic investigations of the *VWF*, including direct sanger DNA sequencing of coding exons, flanking intronic region (~50 base pairs (bp)), promoter, 5′-untranslated region (UTR), and 3′-UTR as well as MLPA, as reported previously [20,27]. In the current study, we further executed NGS analysis to inspect the entire 176 kbp *VWF* which includes all 52 exons, introns (to detect deep intronic pathogenic variants), and regulatory sequences. The computational analysis of the NGS data disclosed a genomic variant call format (VCF) including 215 homozygous high-quality variants, including single-nucleotide variants (SNVs), and small insertion and deletion calls. Consequently, the bioinformatic tool Ensemble Variant Effect Predictor (VEP) demonstrated that 214 out of 215 detected variants (except for variant of c.997 + 118 T > G (g.6073501 A > C)) were previously existing in gene variant database sources (e.g., 1000 Genomes project/dbSNP), and they were not predicted to be deleterious, by exploiting bioinformatic prediction tools Polyphen-2 and SIFT (only for exonic variants), besides SpliceAl (Illumina artificial intelligence splicing prediction software) (Tables S1 and S2). The only exception was a deep intronic variant c.997 + 118 T > G (g.6073501 A > C; located in intron 8 of *VWF*) (Figure 1a). The VEP SpliceAl tool predicted the intronic variant c.997 + 118 T > G can be deleterious, by inducing gain of a donor ss, providing the delta score for donor gain of 0.95 (the SpliceAl delta Score ranges from 0 to 1, and the higher the score, the higher the possibility of splice-altering the variant) (Figure 1a). The suggested SpliceAl cutoffs are 0.2 (high recall), 0.5 (recommended), and 0.8 (high precision). Further, to ascertain the consequence of the intronic DNA variant, we additionally utilized splicing prediction bioinformatics programs of Neural Network Splicing, Alternative Splice Site Predictor (ASSP), and plug-in MaxEnt (for 5′ donor site) of the Human Splicing Finder (HSF). Similarly, these splicing prediction tools predicted that the detected intronic variant 997 + 118 T > G can create a new donor splice site in intron 8 of *VWF* (Figure 1b). The bioinformatics tools demonstrated that this deep *VWF* variant creates a splice site, which could outcompete the original consensus 5′ donor ss at exon/intron 8 junction (Figure 1c). For example, the ASSP tool presented a prediction score of 10.820 for the variant c.997 + 118 T > G, versus (vs.) no ss-prediction for the native residue (c.997 + 118 T) and vs. prediction score of 8.516 for the consensus 5′ donor ss (Figure 1b). Likewise, the prediction tools MaxEnt and Neural Network Splicing provided figures (as shown in Figure 1b) verifying that the variant 997 + 118 T > G in the deep intron 8 of *VWF* could override the original consensus 5′ donor ss. This leads to an aberrant mRNA that comprises partial intron 8 (first 118 nucleotides, called a pseudoexon), containing a premature stop codon, which would make this aberrant mRNA susceptible to nonsense-mediated mRNA decay (NMD) (Figure 1c).

**Table 1:** Phenotypic characteristics and genetic data of the index patient.

	Mutation Routine DNA Analysis (Sanger Seq. and MLPA)	Mutation NGS of Whole *VWF*	Age (Years)	VWF:Ag IU/dL	VWF:GPIbM IU/dL	FVIII:C IU/dL	Bleeding Manifestations
**Index Patient**	No mutation	c.997 + 118 T > G (g.6073501 A > C)	26	<4	<4	2.0	Frequent nose bleeding, easy bruising, menorrhagia, bleeding after injury
**Normal Range**	―	―	―	65–165	64–150	70–157	―

Seq, sequencing; NGS, next-generation sequencing; *VWF*, VWF gene; VWF:Ag, von Willebrand factor antigen; VWF:GPIbM, mutant GPIb (glycoprotein Ib) binding activity of VWF; FVIII:C, factor VIII activity.

### 2.2. ECFC Characterization: Confirming Endothelial Cells’ Phenotype

**Figure 2:**
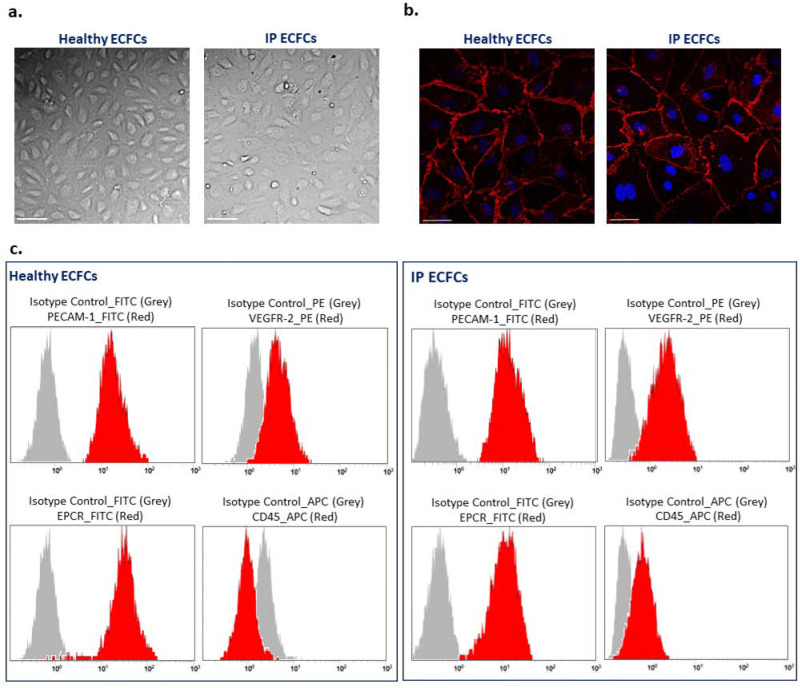
Phenotype characterization of endothelial colony-forming cells (ECFCs). (**a**) Typical endothelial cobblestone-like morphology of ECFCs isolated from a healthy individual and the index patient (IP) observed using bright-field microscopy. Scale bars, 100 µm. (**b**) Expression of VE-cadherin (red) at cell–cell junctions is visualized with immunostaining of ECFCs derived from a healthy individual (left side) and patient (IP ECFCs) (right side). The nucleus is stained with DAPI (blue). Scale bars, 50 µm. (**c**) Flow cytometry analysis of ECFCs derived from a healthy donor (left images) and the IP (right images). The data confirmed that both healthy and IP isolated cells were positive for endothelial cell canonical markers of PECAM-1 (CD31-FITC conjugated), VEGFR-2 (PE-conjugate), and EPCR (FITC-conjugate); besides, they were negative for leukocyte cell marker CD45 (APC-conjugate). Isotype controls were conjugated with either FITC, PE, or APC corresponding to their relevant antibodies, and they are shown as grey bell curve graphs.

The IP and healthy ECFCs isolated and expanded in culture displayed classic endothelial cobblestone morphology (Figure 2a). Further, the ECFCs exhibited canonical endothelial cell surface markers after fluorescence microscopy or flow cytometry analysis, validating the endothelial nature of ECFCs (Figure 2b,c). The immunofluorescence microscopy demonstrated the presence of VE-cadherin at cell–cell junctions in both IP and healthy ECFCs (Figure 2b). Further characterization by flow cytometry confirmed that IP-derived and healthy ECFCs expressed endothelial markers CD31 (PECAM-1), VEGFR-2, and EPCR, and they were negative for the monocyte marker CD45 (Figure 2c).

### 2.3. VWF RNA Transcripts, Endogenous VWF Biosynthesis, and Storage in ECFCs

**Figure 3:**
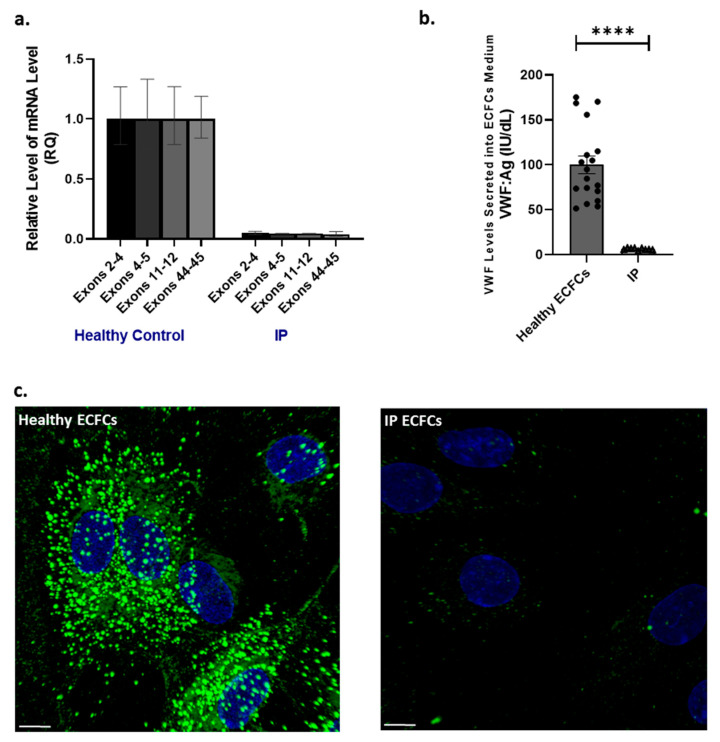
Von Willebrand factor (VWF) mRNA expression, as well as VWF secretion and intracellular storage, in endothelial colony-forming cells (ECFCs). (**a**) Illustration of comparative VWF mRNA levels in the index patient (IP) ECFCs quantified by real-time PCR, using primer/probe combinations directing four different sites in VWF cDNA, across exons 2–4, 4–5, 11–12, and 43–45. The measurements were performed based on the comparative C_T_(∆∆ C_T_) method. Measurements of VWF mRNA levels were normalized to endogenous glyceraldehyde-3-phosphate dehydrogenase (GAPDH) or actin beta (ACTB) mRNA. (**b**) Graph of the mean of VWF antigen (VWF:Ag) levels in the medium of ECFCs obtained from IP (three independent ECFC isolations, three different passages each; N = 9) and six healthy donors (three different passages each; N = 18). (**c**) Immunofluorescence images of ECFCs isolated from the IP and healthy individuals. In healthy ECFCs, VWF (green) is deposited in stick-shaped organelles, resembling Weibel–Palade bodies (WPBs). However, VWF staining in the IP-ECFCs showed almost a lack of WPB formation, except for a few WPBs (1 to 3) in some cells. The scale bar is 10 µm.

Quantitative VWF transcription analysis of the IP-derived ECFCs by real-time PCR, using four primer/probe sets targeting different sequences through *VWF*, showed a significant reduction in VWF mRNA (very low VWF mRNA, only a trace of VWF mRNA detected) (Figure 3a). The obtained mean of relative quantity (RQ) of VWF mRNA levels in IP ECFCs was 0.05, 0.05, 0.05, and 0.04 once the primers and probes were directing sequences spanning exons 2–4, 4–5, 11–12, and 43–45, respectively (vs. RQ of 1.00 for the VWF mRNA in healthy ECFCs) (Figure 3a). Furthermore, the cDNA sequencing demonstrated that the detected small trace of mRNA in IP ECFCs was intact, and no aberrant VWF mRNA was detected. These results support the bioinformatics tool predictions which point to NMD degradation of the aberrant VWF mRNA (containing pseudoexon). Although we do not have actual experimental proof of the altered spicing, the absence of aberrant transcript is mostly indicative of a disease-causing variant that disrupts normal splicing and targets abnormal mRNAs for degradation.

Further, the levels of VWF:Ag (IU/dL) secreted into the supernatant of healthy and IP ECFCs were measured, and the IP-ECFC VWF levels (average of three cell passages for each of three different cultures, N = 9) are here presented as a percentage of the mean value of VWF levels in healthy ECFC supernatant (average of six healthy donor ECFCs, three cell passages each, N = 18). In keeping with VWF mRNA transcripts levels, the secreted VWF:Ag levels from the IP-ECFCs were significantly diminished (6.2% ± 0.4 of healthy controls, *p* < 0.0001) (Figure 3b). Accordingly, immunofluorescence microscopy of the healthy ECFCs indicated that the majority of VWF staining appeared as stick-like structures, signifying their storage in WPBs. However, the IP ECFCs were almost devoid of WPBs; only a few (one to three) small round WPBs were detected in some ECFCs (Figure 3c).

### 2.4. Alternative Trafficking of WPBs’ Proinflammatory Ang2 Cargo in IP Cells

**Figure 4:**
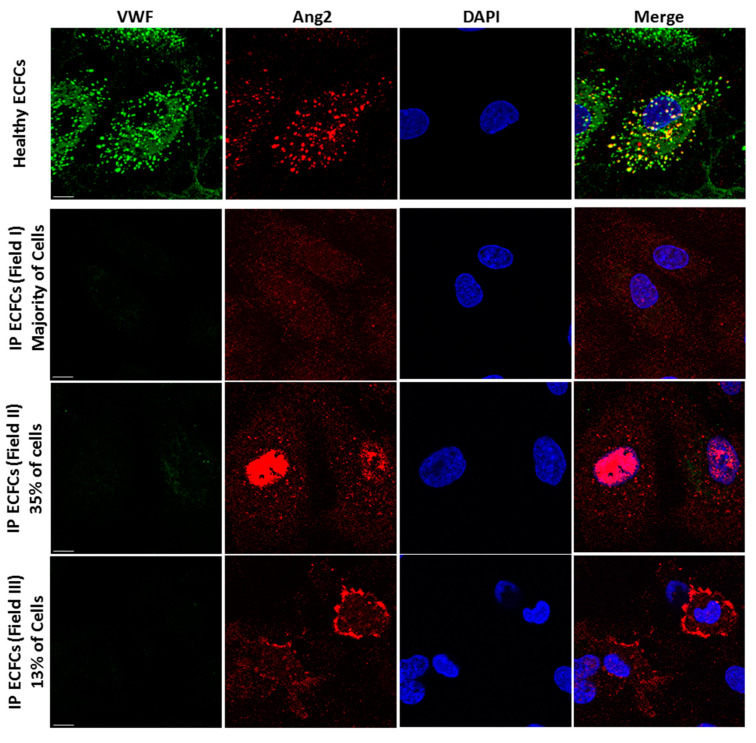
**Altered trafficking of angiopoietin-2 (Ang2), the inflammatory cargo of Weibel–Palade bodies (WPBs), in index patient (IP) endothelial colony-forming cells (ECFCs)**. Storage of VWF (green) and Ang2 (red) in WPBs is visualized by immunofluorescence staining and subsequent microscopy analysis (by Zeiss Apotome.2 microscopy) of normal ECFCs (upper section). The merge of green and red channels displays the colocalization of VWF with Ang2 in healthy ECFCs. In the IP ECFCs, in absence of VWF and WPBs, the storage of Ang2 is changed. In the majority of IP ECFCs, the Ang2 is spread throughout the cytoplasm (the second row of images); in about 35% of IP cells, the Ang2 is accumulated nearby the nucleus (third row); and in about 13% of IP cells, the Ang2 signals are enriched at the cell periphery (lower row images). Scale bars, 10 µm.

We additionally explored the intracellular fate and trafficking of inflammatory cargo of WPBs, Ang2, in the (virtual) absence of VWF biosynthesis and lack of WPB formation, by immunofluorescence microscopy. Immunostaining of healthy ECFCs and subsequent visual inspection of cells with Apotome.2 microscopy exhibited that Ang2 principally appears as punctate structures, and it is mostly colocalized with VWF in WPBs (Figure 4). Additionally, in about 10% of healthy ECFCs, a few Ang2 signals were detected in the nucleus. Alternatively, in IP ECFCs (in absence of VWF and WPBs), Ang2 configuration was different from what we observed in healthy ECFCs, and the intracellular trafficking of Ang2 was altered as well. In IP ECFCs, the Ang2 appeared as small particles rather than punctate structures observed in healthy ECFCs. The Ang2 was distributed throughout the cytoplasm of the majority of IP cells; additionally, 35% of cells exhibited substantial accumulation of Ang2 to the district nearby the nucleus (but not inside the nucleus). Besides, in some of the IP ECFCs (about 13%), the Ang2 was accumulated in the cell periphery (Figure 4). 

### 2.5. Differential Gene Expression and Altered Signaling Pathways in IP ECFCs

**Figure 5:**
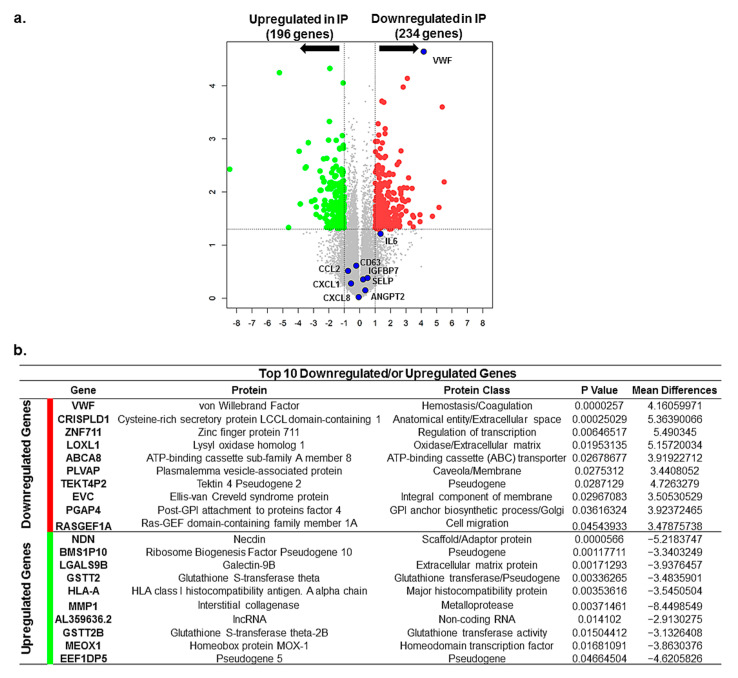
**Differentially expressed genes (DEGs) and the top 10 downregulated or upregulated genes in index patient (IP) endothelial colony-forming cells (ECFCs)**. (**a**) Volcano plot demonstrates significantly DEGs in IP ECFCs in which –log10 (*p*) is plotted against the mean differences. Green dots represent the upregulated genes and red dots represent downregulated genes. Numbers of genes upregulated or downregulated are indicated. Dashed lines represent the threshold of statistical significance. DEGs were considered significant when *p*-value < 0.05 and fold change was greater than 2 with respect to healthy ECFC samples or absolute log2FC (log2 fold change or called mean difference) was more than 1. A negative mean difference value points to lower expression in healthy controls (upregulated expression in IP), and a positive mean difference indicates lower expression in IP-ECFCs. The blue dots illustrate the expression of the VWF, the main Weibel–Palade body (WPB) cargo, which is significantly downregulated, as well as expression of inflammatory cargos of WPBs (Ang2, CCL2, CD63, IGFBP7, CXCL8, IL6, CXCL1, and SELP), which does not show any changes compared with healthy ECFCs. Ang2 (ANGP2), angiopoietin-2; CCL2 (known as MCP-1), C-C motif chemokine ligand 2; IGFBP7, insulin-like growth factor binding protein 7; CXCL8 (known as interleukin 8 (IL8)), C-X-C motif chemokine ligand 8; IL6, interleukin 6; CXCL1 (knows as GROa), C-X-C motif chemokine ligand 1; SELP, P-selectin. (**b**) The table presents the top ten downregulated as well as top ten upregulated genes in the ECFCs isolated from the patient. The list is generated according to the mean difference (absolute log2FC), and they are sorted by decreasing *p*-value in the present table.

We explored whether the lack of VWF and WPBs has in particular affected the expression level of inflammatory proteins stored in WPBs and whether lack of WPBs and altered trafficking of WPBs’ inflammatory cargo Ang2 have induced changes in overall gene expression of ECFCs. To address this vagueness, we compared gene expression profiling of two different healthy ECFCs (two cell passages each, N = 4) with the IP ECFCs (two different colonies isolated from the patient, N = 2) after completing the whole-transcriptome RNA-sequencing (RNA-seq). From a total of 18,912 detected mRNAs, there were 430 differentially expressed genes (DEGs, including 196 upregulated and 234 downregulated genes) in IP ECFCs, compared to the healthy individuals (Figure 5a). As expected, the RNA-seq data showed a substantial reduction in VWF mRNA levels in IP ECFCs compared with healthy ECFCs, confirming the data obtained from quantitative real-time PCR. Nevertheless, the RNA-seq data did not reveal deviations in the expression of WPBs’ inflammatory cargos (Ang2, P-selectin (SELP), IL-6, IL8, GROα (CXCL1), CCL2 (MCP1), CD63, and IGFBP7) in IP ECFCs, when it is compared with healthy cells (Figure 5a).

The top 10 downregulated and upregulated genes (top 20 DEGs), according to mean difference (absolute log2FC (fold change)), are presented in Figure 5b, sorted by decreasing *p*-value. 

## 3. Discussion

In the current study, we sequenced the entire 176 kbp VWF of a type 3 VWD IP using the NGS strategy to scan deep intronic regions, after conventional diagnostic genetic testing (Sanger sequencing and MLPA) failed to identify the genetic cause of the disease. Our NGS analysis revealed a novel homozygous substitution (c.997 + 118 T > G) deep in intron 8 of VWF. The variant was predicted by several bioinformatics tools to create a new donor ss (overriding the consensus 5′ donor ss at exon/intron 8 junction), which leads to the generation of an aberrant transcript with an insertion of the first 118 nt of intron 8 (as a pseudoexon). This transcript, including the pseudoexon, disrupts the reading frame and consequently introduces a premature stop codon, targeting the aberrant mRNA for degradation by NMD. Consistently, the virtual absence of VWF mRNA (except a trace of normal transcript) was validated by subsequent RT-PCR and RNA-seq analyses. These data are in agreement with almost no VWF production in IP-derived endothelial cells (ECFCs). Only a residual amount of the VWF was secreted into the supernatant of the IP-derived ECFCs (VWF:Ag levels of 6.2% of healthy individuals), and a very minor VWF signal in IP ECFCs was observed by the immunofluorescence microscopy inspections. Indeed, a residual amount of normal VWF transcript, even in the occurrence of the splicing intronic variant (c.997 + 118 T > G), suggests that this variant behaves as a leaky mutation. To our knowledge, here for the first time, we report a deep intronic substitution, leading to mis-splicing, in VWD. According to the Human Gene Mutation Database (HGMD), approximately 10% of the reported VWF variants are splicing mutations. However, this number might be undervalued due to the lack of transcript assays. The previously reported VWD-associated splicing mutations occur within or close to the conserved consensus 5′ donor or 3′ ss (e.g., c.323 + 1 G > T, or c.2547−13 T > A), as well as exonic modulatory splicing elements, which cause either exon skipping or intron retention [30,31,32,33,34]. All these VWD-causing splicing mutations were detected by Sanger sequencing or targeted NGS, which cover exons and exon–intron junctions (usually up to 50 bp of introns). In the present study, for the first time, we applied whole-genome NGS to search for a causative mutation and consequently suggested the NGS of the whole VWF genomic region as a plausible alternative for detecting rare mutations whenever the conventional diagnostic genetic testing is unraveling. 

Nonetheless, in very recent years, with the advent of NGS technology, deleterious DNA variants in deep intronic regions have been increasingly described in multiple diseases [35]. The deep intronic variants, causing generation of a new donor ss (leading to pseudoexon inclusion), have been previously reported in many monogenic disorders such as Usher syndrome, nemaline myopathy, Gaucher disease, hemophilia, hereditary angioedema, inherited retinal diseases (a major cause of inherited blindness), and Alport syndrome (hereditary nephritis), as well as hereditary cancer syndromes [36,37,38,39,40,41,42]. The incidence of pseudoexon inclusion (due to the intronic mutations) is now considered a more common cause of inherited disease than formerly thought [35]. Vaz-Drago et al. has gathered the evidence of occurring mutations within introns of over 75 inherited diseases in their review, and they have reported that the deep intronic mutations, imbedded more than 100 bp away from exon–intron junctions, most commonly lead to pseudoexon inclusion due to activation of noncanonical ss or changes in splicing regulatory sequences [35]. Further, they have described that in the majority of the cases with the deep intronic mutation and appearance of a pseudoexon, the mutant mRNA species are degraded by NMD due to introducing a premature termination codon. Our data in the present study, in accord with these previous studies, highlight the importance of the NGS (scanning of the extraexonic region) combined with the mRNA transcript analysis and investigation of protein production in patient-derived cells for the genetic diagnosis of patients bearing no causative mutation in their exonic coding regions.

VWF is a prerequisite for the formation of its storage vesicles, WPBs, proved by the lack of WPBs in ECFCs of a previously reported type 3 VWD patient as well as the current type 3 IP VWD, or VWF knock-out animal models [16,43,44,45,46,47]. In the current study, we evaluated intracellular trafficking of Ang2, the inflammatory/angiogenesis cargo of WPBs, in the absence of VWF and WPBs. The IP ECFCs demonstrated changes in intracellular trafficking and organization of Ang2, illustrating distribution throughout the cell cytoplasm, which is different from healthy ECFCs (with punctate-like structures, colocalized with VWF inside WPBs). Furthermore, in IP ECFCs, Ang2 was also accumulated either around the cell nucleus (35% of cells) or at the cell periphery (13% of cells). Interestingly, very recently, we reported nuclear accumulation of Ang2 in ECFCs of another type 3 VWD with a heterozygous large deletion of *VWF* exons 4–34 [48]. In this patient, deleted VWF protein had a dominant-negative impact on the elongation of multimers and the biogenesis of WPBs. In this formerly described patient, Ang2 signals appeared cloudy/smeary, and they were relocated substantially inside the nucleus in the majority of cells (in more than 70% of cells). However, in the current type 3 IP VWD patient, the Ang2 signals were more discrete/particulate, and in about one-third of cells, they were only distinctly accumulated nearby the nucleus (and not inside the nucleus). It seemed though Ang2 molecules are dragged towards the nucleus, they cannot cross the nucleus due to their size. Although the critical roles of Ang2 in inflammation and angiogenesis are mainly defined through Tie2 on the endothelial surface, a nuclear accumulation of Ang2 following stimulation of healthy endothelial cells has been reported [49,50,51,52]. This evidence indicates the potential roles of Ang2 in the nucleus. Nevertheless, future extensive investigations remain to be conducted to understand the role of intact VWF in the organization and trafficking of Ang2. Furthermore, of interest, MMP1 (interstitial collagenase), with functions in inflammation and angiogenesis, is among the top 10 upregulated genes in both our currently and previously reported VWD patients, as well as the CRISPR/Cas9-engineered VWF-deficient ECFCs, indicating the significance of impaired VWF/WPBs on overall protein expression in endothelial cells (Schillemans et al., 2019). 

In conclusion, this study highlights the importance of NGS analysis for scanning the deep intronic regions combined with mRNA analysis and in-depth investigation of patient-derived endothelial cells for identification and validation of deleterious DNA variants in the type 3 VWD patients without any mutation in their exonic regions. Furthermore, our findings demonstrated that in the absence of VWF and WPBs, the intracellular trafficking and storage arrangement of WPBs’ inflammatory molecule Ang2 was altered.

## 4. Materials and Methods

### 4.1. Patient: Phenotype and Genetic Analyses

A 26-year-old female index patient (IP) with VWD was recruited in the current study. 

ISTH-BAT questionnaire was administered to record patient bleeding history [53]. The IP comes from a genetically isolated population, suggesting a high level of homozygosity in the population. 

NGS using Illumina Beadarrays was executed to analyze the entire 176 kbp *VWF* which includes all 52 exons, introns (to detect deep intronic pathogenic variants), and promoter region. Total genomic DNA was isolated from patient EDTA blood using the standard salting-out procedure. Sequencing libraries were generated using an Illumina TruSeq DNA PCR-Free Library Preparation Kit (Illumina, Cambridge, UK). After quality assessments, the libraries were sequenced on an Illumina platform (NovaSeq 6000) at 30× coverage using 2 × 150 bp reads. After importing an obtained FASTQ file into the CLC Genomics Workbench (Qiagen, Hilden, Germany), the reads were aligned to the human reference chromosome 12 (chr12 NC000012). Subsequently, the screening of the genomic variants residing in *VWF* (chr12: 5,948,877-6,124,770, according to Genome Reference Consortium Human Build 38 patch release 13 (GRCh38.p13)) was executed. The homozygous variants, including SNVs and small insertion and deletion calls, at a minimum coverage of 10× and allele frequency of 92% or higher were called. Consequently, the variant annotation and pathogenic effect of the all homozygous SNVs were predicted by the Ensemble VEP (https://www.ensembl.org/info/docs/tools/vep/index.html, accessed on 8 April 2021), which indicates the location of the variant (e.g., upstream a transcript, in the coding sequence, in the intronic region, or in the regulatory region), evaluates if a variant is known (associated with minor allele frequency from the 1000 Genomes project/dbSNP), and determines the pathogenic effect of the variants using bioinformatic prediction tools such as Polyphen-2, SIFT, and SpliceAl [54,55]. Further, the splicing prediction tools Neural Network Splicing (https://www.fruitfly.org/seq_tools/splice.html, accessed on 9 December 2021), ASSP (http://wangcomputing.com/assp/index.html, accessed on 9 December 2021), and HSF-MaxEnt donor site plug-in (https://www.genomnis.com/the-system-1, accessed on 10 December 2021) were utilized to approve the predicted variant consequence after VEP SpliceAl [56,57].

### 4.2. Isolation and Ex Vivo Expansion of ECFCS

ECFCs were obtained from the peripheral blood of the IP and six healthy donors based on the published standardized protocols [33,58]. The endothelial phenotype of the ECFCs was confirmed by flow cytometry using REAfinity fluorochrome-conjugated antibodies IgG1 (Miltenyi Biotec, Bergisch Gladbach, Germany) directed against endothelial surface markers CD31 (PECAM-1), CD309 (VEGFR-2), and CD201 (EPCR), besides hematopoietic marker CD45, based on the standard approaches [59], as well as detecting cell surface marker VE-cadherin by immunofluorescence microscopy, as described in next section. To accomplish flow cytometry analysis, the confluent expanded ECFCs were trypsinized and were resuspended at a concentration of 10^6^ cells per mL in staining buffer (PBS with 2% FBS and 2 mM EDTA). Subsequently, cells were stained with fluorescently conjugated isotype control antibodies or antibodies directed against specific surface markers. The cytometric analysis was completed on Navios Flow Cytometer (Beckman Coulter, Krefeld, Germany).

### 4.3. VWF Transcript Analysis and Quantitative Real-Time PCR

Total RNA from peripheral blood and ECFCs was extracted from the patient and healthy individuals using the Tempus Spin RNA isolation kit (Applied Biosystems, Knutsford, UK) and RNeasy Mini kit (Qiagen, Hilden, Germany), respectively. The reverse transcription (RT) and amplification of the full-length VWF cDNA were executed in 10 overlapping fragments using the Qiagen LongRange 2Step RT-PCR kit (Qiagen, Hilden, Germany). The RT-PCR products were electrophoresed on 1% agarose gel and subsequently sequenced, as described previously [33]. 

The quantification of the VWF mRNA in ECFCs was completed using the TaqMan assay on an ABI 7500 real-time PCR system (Applied Biosystems, Waltham, MA, USA). The quantitative RT-PCR was performed using TaqMan Reverse Transcription Reagents and TaqMan Universal PCR Master Mix (Applied Biosystems, Waltham, MA, USA), as well as using four sets of fluorogenic probe/primer combinations directing various sites in VWF cDNA. In the first set, the forward primer targeted a sequence in exon 2, and the probe and reverse primer were both designed to target sequences in exon 3 of VWF. In the second set, the forward primer and the probe both were designed to target sequences in exon 4, and a reverse primer was designed across the exons 4–5 junction of VWF cDNA. In the third set, the forward and the reverse primers were directed at exons 11 and 12, respectively, and the fluorogenic probe targeted a sequence across the exons 11–12 junction. The fluorogenic probe, forward primer, and reverse primer targeted sequences in exon 44, exons 43–44, and exons 44–45 junctions, respectively, in the fourth set. The VWF mRNA levels were normalized to endogenous glyceraldehyde-3-phosphate dehydrogenase (GAPDH) or actin beta (ACTB) mRNA (both provided by Applied Biosystems). The acquired numbers were assessed by 7500 software v2.0.6 based on the comparative C_T_(∆∆ C_T_) approach. 

### 4.4. Whole-Transcriptome RNA-Seq Analysis

Total RNA isolated from ECFCs of two healthy adult individuals (every 2 samples, N = 4) and the IP (2 samples) were subjected to the high-throughput RNA-seq. After quantity and quality evaluations, RNA-seq libraries were generated using QuantSeq 3’-mRNA Library Prep kit (Lexogen, Wien, Austria) and subsequently sequenced on an Illumina HiSeq 2500 V4 platform (Illumina, San Diego, CA, USA). After quality assessment and trimming tasks, the obtained reads were aligned to the reference human genome GrCH38.8 (Ensemble) using the miARMa pipeline [60,61,62]. Tophat2 and bowtie2 were used to complete mapping [61,63]. The reads were converted to FPKM (fragments per kilobase of transcript per million mapped reads), and the additional quality control was performed via Qlucore Omics Explorer 3.6 (Qlucore, Lund, Sweden). The genes with *p*-value ≤ 0.05 and fold change ≥2 (or a mean difference (absolute log2FC) > 1) were considered statistically significant DEGs. 

### 4.5. VWF Production Evaluation in ECFCs

The VWF levels secreted into the supernatant media and present in the cell lysates of the six healthy ECFCs (three different samples each; N = 18) and IP-ECFCs (six samples from various cell passages, the same as the passage numbers of the healthy individuals; N = 6) were determined. Seventy-two hours after seeding cells (at a density of 1.5 × 10^6^ cells/10 mL in 75 cm^2^ flasks), the supernatant medium was collected, and cells were lysed. VWF present in media and cell lysates was concentrated on Amicon centrifugal filter devices (Millipore, Burlington, MA, USA), VWF:Ag levels were measured, and VWF multimers were analyzed by electrophoresis on 1.2% and 1.6% SDS-agarose gel [33,64].

### 4.6. Immunofluorescence Microscopy

Confluent ECFCs were fixed, permeabilized, and stained as previously described in detail [33]. To visualize VWF and VE-cadherin (endothelial adhesion molecules at cell–cell junctions), as well as WPBs’ inflammatory molecule Ang2, the polyclonal rabbit anti-human VWF (DAKO, Glostrup, Denmark) or sheep anti-human VWF (Abcam, Cambridge, UK), anti-VE-cadherin (Santa Cruz Biotechnology, Heidelberg, Germany), and monoclonal mouse anti-Ang2 (F-1; Santa Cruz Biotechnology, Santa Cruz, CA, USA) were used to immunostain the ECFCs. Imaging of the cells was carried out using an Apotome.2 microscope (Carl Zeiss, Cologne, Germany). Three-dimensional (3D) images of piled-up z-stacks were generated by the ZEN 2.6 program (blue edition; Carl Zeiss, Cologne, Germany).

### 4.7. Data Analysis

All data are expressed as mean ± SEM. Statistical significance of the results was examined by unpaired Student’s *t*-test using GraphPad Prism version 8.0.1 (GraphPad Software, San Diego, CA, USA). A *p*-value of <0.05 was considered statistically significant.

## Data Availability

The raw RNA-seq datasets presented in this study can be found in the online repository NCBI-GEO, under the accession number GSE195695.

## Supplementary Materials

The following supporting information can be downloaded at: https://www.mdpi.com/article/10.3390/ijms23063095/s1.
