# Supplementary material for: A Homozygous Deep Intronic Variant Causes Von Willebrand Factor Deficiency and Lack of Endothelial-Specific Secretory Organelles, Weibel–Palade Bodies
Source: Int J Mol Sci. 2022 Mar 13;23(6):3095. doi: 10.3390/ijms23063095 (PMC8950443; doi:10.3390/ijms23063095)
Supplement: Supplementary file 1 [file ijms-23-03095-s001.zip › ijms-1603148-supplementary.pdf]

Supplementary Materials: A homozygous deep intronic variant causing von Willebrand factor deficiency and lack of endothelial-specific secretory organelles, Weible-Palade bodies

Table S1. List of homozygous variants located in entire von Willebrand factor (VWF) gene of the index patient, detected by next-generation sequencing, and subsequent bioinformatics analysis by Ensemble Variant Effect Predictor (VEP)

	Location	Allele	Consequence	SYMBOL	Existing variation	SIFT	PolyPhen	CLIN_SIG	SpliceAI_pred DS_AG	SpliceAI_pred DS_AL	SpliceAI_pred DS_DG	SpliceAI_pred DS_DL
**1**	NC_000012:6121122-6121122	G	intron_variant	VWF	rs6489694	-	-	-	0.00	0.00	0.00	0.00
**2**	NC_000012:6116076-6116076	G	intron_variant	VWF	rs4764537	-	-	-	0.00	0.00	0.00	0.00
**3**	NC_000012:6107670-6107670	T	intron_variant	VWF	rs4485164	-	-	-	0.00	0.00	0.00	0.00
**4**	NC_000012:6095736-6095737	-	intron_variant	VWF	rs5796209	-	-	-	0.00	0.00	0.00	0.00
**5**	NC_000012:6093442-6093442	A	intron_variant	VWF	rs2058223	-	-	-	0.00	0.00	0.00	0.00
**6**	NC_000012:6093296-6093296	G	intron_variant	VWF	rs12317079	-	-	-	0.00	0.00	0.00	0.00
**7**	NC_000012:6092634-6092634	A	intron_variant	VWF	rs142695490	-	-	-	0.00	0.00	0.00	0.00
**8**	NC_000012:6092632-6092632	A	intron_variant	VWF	rs148892733	-	-	-	0.00	0.00	0.00	0.00
**9**	NC_000012:6092630-6092630	A	intron_variant	VWF	rs143663161	-	-	-	0.00	0.00	0.00	0.00
**10**	NC_000012:6092628-6092628	A	intron_variant	VWF	rs144959320	-	-	-	0.00	0.00	0.00	0.00
**11**	NC_000012:6092626-6092626	A	intron_variant	VWF	rs150759772	-	-	-	0.00	0.00	0.00	0.00
**12**	NC_000012:6092624-6092624	A	intron_variant	VWF	rs144868844	-	-	-	0.00	0.00	0.00	0.00
**13**	NC_000012:6092622-6092622	A	intron_variant	VWF	rs145245519	-	-	-	0.00	0.00	0.00	0.00
**14**	NC_000012:6092620-6092620	A	intron_variant	VWF	rs147129737	-	-	-	0.00	0.00	0.00	0.00
**15**	NC_000012:6092519-6092519	G	intron_variant	VWF	rs3063460	-	-	-	0.00	0.00	0.00	0.00
**16**	NC_000012:6092515-6092515	TG	intron_variant	VWF	rs66551998	-	-	-	-	-	-	-
**17**	NC_000012:6092028-6092028	G	intron_variant	VWF	rs2239139	-	-	-	0.00	0.00	0.00	0.00
**18**	NC_000012:6091277-6091277	T	intron_variant	VWF	rs2239140	-	-	-	0.00	0.00	0.00	0.00
**19**	NC_000012:6089912-6089912	C	intron_variant	VWF	rs2239142	-	-	-	0.00	0.00	0.00	0.00
**20**	NC_000012:6089774-6089774	G	intron_variant	VWF	rs1034933	-	-	-	0.00	0.00	0.00	0.00
**21**	NC_000012:6089557-6089557	T	intron_variant	VWF	rs2109119	-	-	-	0.00	0.00	0.00	0.00

**22**	NC_000012:6089081-6089081	T	intron_variant	VWF	rs10849382	-	-	-	0.00	0.00	0.00	0.00
**23**	NC_000012:6089075-6089075	T	intron_variant	VWF	rs10849381	-	-	-	0.00	0.00	0.00	0.00
**24**	NC_000012:6089065-6089065	G	intron_variant, non_coding transcript_variant	VWF	rs2238101	-	-	-	0.00	0.00	0.00	0.00
**25**	NC_000012:6088302-6088302	C	intron_variant	VWF	rs2238102	-	-	-	0.00	0.00	0.00	0.00
**26**	NC_000012:6085489-6085489	C	intron_variant	VWF	rs2109118	-	-	-	0.00	0.00	0.00	0.00
**27**	NC_000012:6085077-6085077	G	intron_variant	VWF	rs1860364	-	-	-	0.00	0.00	0.00	0.00
**28**	NC_000012:6085017-6085017	A	intron_variant	VWF	rs2238103	-	-	-	0.00	0.00	0.01	0.00
**29**	NC_000012:6084371-6084371	G	intron_variant	VWF	rs763580	-	-	-	0.00	0.00	0.00	0.00
**30**	NC_000012:6083930-6083930	T	intron_variant	VWF	rs3819537	-	-	-	0.00	0.00	0.00	0.00
**31**	NC_000012:6083720-6083720	G	intron_variant	VWF	rs3819538	-	-	-	0.00	0.00	0.00	0.00
**32**	NC_000012:6081339-6081339	T	intron_variant	VWF	rs7133854	-	-	-	0.00	0.00	0.00	0.00
**33**	NC_000012:6073501-6073501	C	intron_variant	VWF	-	-	-	-	0.00	0.00	**0.95**	0.00
**34**	NC_000012:6070224-6070224	T	intron_variant	VWF	rs137873960	-	-	-	0.00	0.00	0.00	0.00
**35**	NC_000012:6066859-6066859	C	intron_variant	VWF	rs2238106	-	-	-	0.00	0.00	0.00	0.00
**36**	NC_000012:6063182-6063182	A	intron_variant	VWF	rs2239159	-	-	-	0.00	0.00	0.00	0.00
**37**	NC_000012:6062336-6062336	G	intron_variant	VWF	rs2191161	-	-	-	0.00	0.00	0.00	0.00
**38**	NC_000012:6060567-6060567	C	intron_variant	VWF	rs4764482	-	-	-	0.01	0.00	0.01	0.00
**39**	NC_000012:6060277-6060277	A	intron_variant	VWF	rs980133	-	-	-	0.00	0.00	0.00	0.00
**40**	NC_000012:6060032-6060032	C	intron_variant	VWF	rs980131	-	-	-	0.00	0.00	0.00	0.00
**41**	NC_000012:6059861-6059861	C	intron_variant	VWF	rs980130	-	-	-	0.00	0.00	0.00	0.00
**42**	NC_000012:6054787-6054787	A	intron_variant	VWF	rs36100099	-	-	-	0.01	0.00	0.00	0.00
**43**	NC_000012:6053686-6053686	C	intron_variant	VWF	rs2283333	-	-	-	0.00	0.00	0.00	0.00
**44**	NC_000012:6053588-6053588	G	intron_variant	VWF	rs2239160	-	-	-	0.00	0.00	0.00	0.00
**45**	NC_000012:6053495-6053495	A	intron_variant	VWF	rs2239161	-	-	-	0.00	0.00	0.00	0.00
**46**	NC_000012:6051577-6051578	TG	intron_variant	VWF	rs386759880	-	-	-	-	-	-	-
**47**	NC_000012:6051526-6051526	A	intron_variant	VWF	rs4764531	-	-	-	0.00	0.00	0.00	0.00
**48**	NC_000012:6051039-6051039	Ins.	intron_variant	VWF	rs75336349	-	-	-	-	-	-	-
**49**	NC_000012:6050980-6050980	C	intron_variant	VWF	rs11064010	-	-	-	0.00	0.00	0.00	0.00
**50**	NC_000012:6050879-6050879	T	intron_variant	VWF	rs10849379	-	-	-	0.01	0.00	0.00	0.00

**51**	NC_000012:6050371-6050371	G	intron_variant	VWF	rs216304	-	-	-	0.00	0.00	0.00	0.00
**52**	NC_000012:6049879-6049879	T	intron_variant	VWF	rs216303	-	-	-	0.00	0.00	0.00	0.00
**53**	NC_000012:6048856-6048856	A	intron_variant	VWF	rs216302	-	-	-	0.00	0.00	0.00	0.00
**54**	NC_000012:6048822-6048822	G	intron_variant	VWF	rs216301	-	-	-	0.00	0.00	0.00	0.00
**55**	NC_000012:6047469-6047469	G	intron_variant	VWF	rs216299	-	-	-	0.00	0.00	0.00	0.00
**56**	NC_000012:6047311-6047311	T	intron_variant	VWF	rs216298	-	-	-	0.00	0.00	0.00	0.00
**57**	NC_000012:6045504-6045504	G	intron_variant	VWF	rs216296	-	-	-	0.00	0.00	0.00	0.00
**58**	NC_000012:6045040-6045040	C	intron_variant	VWF	rs216295	-	-	-	0.00	0.00	0.00	0.00
**59**	NC_000012:6044584-6044584	G	intron_variant	VWF	rs216294	-	-	-	0.00	0.00	0.00	0.00
**60**	NC_000012:6044493-6044493	T	intron_variant	VWF	rs216293	-	-	benign	0.01	0.00	0.00	0.00
**61**	NC_000012:6044098-6044098	G	intron_variant	VWF	rs11064008	-	-	-	0.00	0.00	0.00	0.00
**62**	NC_000012:6043810-6043810	A	intron_variant	VWF	rs216292	-	-	-	0.00	0.00	0.00	0.00
**63**	NC_000012:6043683-6043683	G	intron_variant	VWF	rs73034873	-	-	-	0.00	0.00	0.00	0.00
**64**	NC_000012:6042707-6042707	T	intron_variant	VWF	rs216291	-	-	-	0.00	0.00	0.00	0.00
**65**	NC_000012:6042431-6042431	A	intron_variant	VWF	rs216290	-	-	-	0.00	0.00	0.00	0.00
**66**	NC_000012:6041658-6041658	A	intron_variant	VWF	rs183356	-	-	-	0.00	0.00	0.00	0.00
**67**	NC_000012:6040118-6040118	C	intron_variant	VWF	rs216339	-	-	-	0.00	0.00	0.00	0.00
**68**	NC_000012:6039160-6039160	G	intron_variant	VWF	rs216338	-	-	-	0.00	0.00	0.00	0.00
**69**	NC_000012:6039143-6039143	C	intron_variant	VWF	rs216337	-	-	-	0.00	0.00	0.00	0.00
**70**	NC_000012:6039064-6039064	G	intron_variant	VWF	rs216336	-	-	-	0.00	0.00	0.00	0.00
**71**	NC_000012:6038839-6038839	C	intron_variant	VWF	rs216335	-	-	-	0.00	0.00	0.00	0.00
**72**	NC_000012:6038445-6038445	T	intron_variant	VWF	rs216334	-	-	-	0.00	0.00	0.00	0.00
**73**	NC_000012:6037953-6037953	C	intron_variant	VWF	rs216333	-	-	-	0.00	0.00	0.00	0.00
**74**	NC_000012:6037890-6037890	A	intron_variant	VWF	rs216332	-	-	-	0.00	0.00	0.00	0.00
**75**	NC_000012:6037848-6037848	T	intron_variant	VWF	rs216331	-	-	-	0.00	0.00	0.00	0.00
**76**	NC_000012:6037464-6037464	C	intron_variant	VWF	rs216330	-	-	-	0.00	0.00	0.01	0.00
**77**	NC_000012:6037341-6037341	C	intron_variant	VWF	rs216329	-	-	-	0.00	0.00	0.02	0.00
**78**	NC_000012:6037317-6037317	T	intron_variant	VWF	rs216328	-	-	-	0.00	0.00	0.00	0.00
**79**	NC_000012:6036983-6036983	C	intron_variant	VWF	rs216327	-	-	-	0.00	0.00	0.00	0.00

**80**	NC_000012:6036782-6036782	A	intron_variant	VWF	rs216326	-	-	-	0.00	0.00	0.00	0.00
**81**	NC_000012:6036363-6036363	A	intron_variant	VWF	rs216325	-	-	benign	0.00	0.00	0.00	0.00
**82**	NC_000012:6036059-6036059	G	intron_variant	VWF	rs216324	-	-	-	0.00	0.00	0.00	0.00
**83**	NC_000012:6035943-6035943	T	intron_variant	VWF	rs216323	-	-	-	0.00	0.00	0.00	0.00
**84**	NC_000012:6035731-6035731	T	intron_variant	VWF	rs216322	-	-	-	0.00	0.00	0.00	0.00
**85**	NC_000012:6035003-6035003	G	intron_variant	VWF	rs669667	-	-	-	0.00	0.00	0.00	0.00
**86**	NC_000012:6034998-6034998	C	intron_variant	VWF	rs499648	-	-	-	0.00	0.00	0.00	0.00
**87**	NC_000012:6034818-6034818	C	missense_variant	VWF	rs216321	Tolerated (1)	Benign (0)	benign	0.00	0.00	0.00	0.00
**88**	NC_000012:6034775-6034775	T	synonymous_variant	VWF	rs756662315	-	-	-	0.00	0.00	0.00	0.00
**89**	NC_000012:6034190-6034190	C	intron_variant	VWF	rs216320	-	-	-	0.00	0.00	0.01	0.00
**90**	NC_000012:6032875-6032875	AC	intron_variant	VWF	rs796891068	-	-	-	-	-	-	-
**91**	NC_000012:6030095-6030095	C	intron_variant	VWF	rs216318	-	-	-	0.00	0.00	0.00	0.00
**92**	NC_000012:6027748-6027748	G	intron_variant	VWF	rs216315	-	-	-	0.00	0.00	0.00	0.00
**93**	NC_000012:6027414-6027414	C	intron_variant	VWF	rs216314	-	-	-	0.00	0.00	0.00	0.00
**94**	NC_000012:6019277-6019277	C	missense_variant	VWF	rs216311	Tolerated (1)	Benign (0)	Benign, likely_benign	0.00	0.00	0.00	0.00
**95**	NC_000012:6018777-6018777	G	synonymous_variant	VWF	rs216310	-	-	likely_benign, benign	0.00	0.00	0.00	0.00
**96**	NC_000012:6017761-6017761	T	intron_variant	VWF	rs216309	-	-	-	0.00	0.00	0.00	0.00
**97**	NC_000012:6015500-6015500	C	intron_variant	VWF	rs216308	-	-	-	0.00	0.00	0.00	0.00
**98**	NC_000012:6011912-6011912	T	intron_variant	VWF	rs216305	-	-	-	0.01	0.00	0.00	0.00
**99**	NC_000012:6009094-6009094	T	intron_variant	VWF	rs216814	-	-	-	0.00	0.00	0.00	0.00
**100**	NC_000012:6008617-6008617	G	intron_variant	VWF	rs216813	-	-	-	0.00	0.00	0.00	0.00
**101**	NC_000012:6005478-6005478	C	intron_variant	VWF	rs216810	-	-	-	0.00	0.00	0.00	0.00
**102**	NC_000012:6004278-6004278	C	intron_variant	VWF	rs216808	-	-	-	0.00	0.00	0.00	0.00
**103**	NC_000012:6003290-6003290	A	intron_variant	VWF	rs216807	-	-	-	0.00	0.00	0.00	0.00
**104**	NC_000012:6002359-6002359	G	intron_variant	VWF	rs216806	-	-	-	0.00	0.00	0.00	0.00
**105**	NC_000012:6002245-6002245	A	intron_variant	VWF	rs216804	-	-	-	0.00	0.00	0.00	0.00
**106**	NC_000012:6000796-6000796	T	intron_variant	VWF	rs188819154	-	-	-	0.00	0.00	0.00	0.00
**107**	NC_000012:6000790-6000792	-	intron_variant	VWF	rs149367153	-	-	-	0.00	0.00	0.00	0.00
**108**	NC_000012:6000736-6000736	C	intron_variant	VWF	rs216803	-	-	-	0.00	0.00	0.00	0.00

**109**	NC_000012:5999937-5999937	A	intron_variant	VWF	rs216802	-	-	-	0.00	0.00	0.00	0.00
**110**	NC_000012:5999347-5999347	A	intron_variant	VWF	rs216801	-	-	-	0.00	0.00	0.00	0.00
**111**	NC_000012:5998780-5998780	T	intron_variant	VWF	rs216800	-	-	-	0.00	0.00	0.00	0.00
**112**	NC_000012:5998451-5998451	C	intron_variant	VWF	rs7398722	-	-	-	0.00	0.00	0.00	0.00
**113**	NC_000012:5996852-5996852	C	intron_variant	VWF	rs216904	-	-	-	0.00	0.00	0.00	0.00
**114**	NC_000012:5996333-5996333	C	intron_variant	VWF	rs216903	-	-	-	0.00	0.00	0.00	0.00
**115**	NC_000012:5996221-5996221	A	synonymous_variant	VWF	rs216902	-	-	likely_benign, benign	0.00	0.00	0.00	0.00
**116**	NC_000012:5995702-5995702	A	intron_variant	VWF	rs216901	-	-	-	0.00	0.00	0.00	0.00
**117**	NC_000012:5994832-5994832	T	intron_variant	VWF	rs12099542	-	-	-	0.00	0.00	0.00	0.00
**118**	NC_000012:5993598-5993598	A	intron_variant	VWF	rs216898	-	-	-	0.00	0.00	0.00	0.00
**119**	NC_000012:5993441-5993441	C	intron_variant	VWF	rs216897	-	-	-	0.00	0.00	0.00	0.00
**120**	NC_000012:5991630-5991630	T	intron_variant	VWF	rs216896	-	-	-	0.00	0.00	0.00	0.00
**121**	NC_000012:5991429-5991429	T	intron_variant	VWF	rs216895	-	-	-	0.00	0.00	0.00	0.00
**122**	NC_000012:5991411-5991411	G	intron_variant	VWF	rs216894	-	-	-	0.00	0.00	0.00	0.00
**123**	NC_000012:5991319-5991319	G	intron_variant	VWF	rs216893	-	-	-	0.00	0.00	0.00	0.00
**124**	NC_000012:5990909-5990909	A	intron_variant	VWF	rs4348960	-	-	-	0.01	0.00	0.00	0.00
**125**	NC_000012:5990862-5990862	A	intron_variant	VWF	rs216892	-	-	-	0.00	0.00	0.00	0.00
**126**	NC_000012:5990683-5990683	G	intron_variant	VWF	rs216891	-	-	-	0.01	0.00	0.00	0.00
**127**	NC_000012:5990372-5990372	C	intron_variant	VWF	rs216890	-	-	-	0.00	0.00	0.00	0.00
**128**	NC_000012:5990287-5990287	C	intron_variant	VWF	rs216889	-	-	-	0.00	0.00	0.00	0.00
**129**	NC_000012:5989691-5989691	G	intron_variant	VWF	rs216888	-	-	-	0.00	0.00	0.00	0.00
**130**	NC_000012:5989286-5989286	C	intron_variant	VWF	rs216887	-	-	-	0.00	0.00	0.00	0.00
**131**	NC_000012:5988799-5988799	T	intron_variant	VWF	rs216886	-	-	-	0.00	0.00	0.00	0.00
**132**	NC_000012:5988789-5988789	C	intron_variant	VWF	rs216885	-	-	-	0.00	0.00	0.00	0.00
**133**	NC_000012:5988662-5988662	A	intron_variant	VWF	rs216884	-	-	-	0.00	0.00	0.00	0.00
**134**	NC_000012:5987650-5987650	T	intron_variant	VWF	rs216882	-	-	-	0.00	0.00	0.00	0.00
**135**	NC_000012:5987546-5987546	G	intron_variant	VWF	rs216881	-	-	-	0.00	0.00	0.00	0.00
**136**	NC_000012:5987525-5987525	A	intron_variant	VWF	rs216880	-	-	-	0.00	0.00	0.00	0.00
**137**	NC_000012:5987510-5987510	A	intron_variant	VWF	rs526881	-	-	-	0.00	0.00	0.00	0.00

**138**	NC_000012:5987507-5987507	G	intron_variant	VWF	rs526861	-	-	-	0.00	0.00	0.00	0.00
**139**	NC_000012:5987489-5987489	T	intron_variant	VWF	rs216879	-	-	-	0.00	0.00	0.00	0.00
**140**	NC_000012:5987217-5987217	C	intron_variant	VWF	rs216878	-	-	-	0.00	0.00	0.00	0.00
**141**	NC_000012:5987128-5987128	C	intron_variant	VWF	rs216877	-	-	-	0.00	0.00	0.00	0.00
**142**	NC_000012:5987090-5987090	A	intron_variant	VWF	rs216876	-	-	-	0.00	0.00	0.00	0.00
**143**	NC_000012:5986735-5986735	G	intron_variant	VWF	rs216875	-	-	-	0.00	0.00	0.00	0.00
**144**	NC_000012:5986641-5986642	TT	intron_variant	VWF	rs71450640	-	-	-	-	-	-	-
**145**	NC_000012:5986591-5986591	C	intron_variant	VWF	rs216874	-	-	-	0.00	0.00	0.00	0.00
**146**	NC_000012:5986108-5986108	C	intron_variant	VWF	rs216873	-	-	-	0.00	0.00	0.00	0.00
**147**	NC_000012:5985679-5985679	A	intron_variant	VWF	rs177702	-	-	benign	0.00	0.00	0.00	0.00
**148**	NC_000012:5984916-5984916	A	intron_variant	VWF	rs24177	-	-	-	0.00	0.00	0.00	0.00
**149**	NC_000012:5984374-5984374	C	intron_variant	VWF	rs216872	-	-	-	0.00	0.00	0.00	0.00
**150**	NC_000012:5983882-5983882	A	intron_variant	VWF	rs216870	-	-	-	0.00	0.00	0.00	0.00
**151**	NC_000012:5982531-5982531	C	intron_variant	VWF	rs190156617	-	-	-	0.00	0.00	0.02	0.00
**152**	NC_000012:5981998-5981998	A	splice_region_variant ,intron_variant	VWF	rs216868	-	-	benign	0.00	0.00	0.00	0.00
**153**	NC_000012:5981834-5981834	G	synonymous_variant	VWF	rs216867	-	-	likely_benign, benign	0.00	0.00	0.00	0.00
**154**	NC_000012:5981137-5981137	C	intron_variant	VWF	rs216865	-	-	-	0.00	0.00	0.00	0.00
**155**	NC_000012:5978365-5978365	C	intron_variant	VWF	rs216859	-	-	-	0.00	0.00	0.00	0.00
**156**	NC_000012:5977875-5977875	A	intron_variant	VWF	rs216858	-	-	-	0.00	0.00	0.00	0.00
**157**	NC_000012:5977770-5977770	T	intron_variant	VWF	rs216857	-	-	-	0.00	0.00	0.00	0.00
**158**	NC_000012:5977099-5977099	C	intron_variant	VWF	rs216856	-	-	-	0.00	0.00	0.00	0.00
**159**	NC_000012:5976347-5976347	G	intron_variant	VWF	rs216855	-	-	-	0.00	0.00	0.00	0.00
**160**	NC_000012:5975698-5975698	C	intron_variant	VWF	rs216854	-	-	-	0.00	0.00	0.00	0.00
**161**	NC_000012:5975547-5975547	A	intron_variant	VWF	rs216853	-	-	-	0.00	0.00	0.00	0.00
**162**	NC_000012:5975157-5975157	G	intron_variant	VWF	rs4764521	-	-	-	0.00	0.00	0.00	0.00
**163**	NC_000012:5975025-5975025	C	intron_variant	VWF	rs216852	-	-	-	0.00	0.00	0.00	0.00
**164**	NC_000012:5973633-5973633	G	intron_variant	VWF	rs12829271	-	-	-	0.00	0.00	0.00	0.00
**165**	NC_000012:5973586-5973586	G	intron_variant	VWF	rs10849366	-	-	-	0.00	0.00	0.00	0.00
**166**	NC_000012:5973392-5973392	T	intron_variant	VWF	rs2239138	-	-	-	0.00	0.00	0.00	0.00

**167**	NC_000012:5972947-5972947	G	intron_variant	VWF	rs917859	-	-	-	0.00	0.00	0.00	0.00
**168**	NC_000012:5972772-5972772	T	intron_variant	VWF	rs917858	-	-	-	0.00	0.00	0.00	0.00
**169**	NC_000012:5971429-5971429	A	intron_variant	VWF	rs11063965	-	-	-	0.00	0.00	0.00	0.00
**170**	NC_000012:5971411-5971411	C	intron_variant	VWF	rs2238110	-	-	-	0.00	0.00	0.00	0.00
**171**	NC_000012:5971368-5971368	C	intron_variant	VWF	rs10849365	-	-	-	0.00	0.00	0.00	0.00
**172**	NC_000012:5971124-5971124	A	intron_variant	VWF	rs7301070	-	-	-	0.02	0.00	0.00	0.00
**173**	NC_000012:5970158-5970158	T	intron_variant	VWF	rs60982029	-	-	-	0.00	0.00	0.00	0.00
**174**	NC_000012:5969892-5969892	T	intron_variant	VWF	rs12300917	-	-	-	0.00	0.00	0.00	0.00
**175**	NC_000012:5969772-5969772	G	intron_variant	VWF	rs11063964	-	-	-	0.00	0.00	0.00	0.00
**176**	NC_000012:5969450-5969450	G	intron_variant	VWF	rs2270239	-	-	-	0.00	0.00	0.00	0.00
**177**	NC_000012:5969258-5969258	T	missense_variant	VWF	rs35335161	Tolerated (0.27)	Benign (0.021)	Benign, likely_benign	0.00	0.00	0.00	0.00
**178**	NC_000012:5968959-5968959	A	intron_variant	VWF	rs4764478	-	-	-	0.00	0.00	0.00	0.00
**179**	NC_000012:5968741-5968741	A	intron_variant	VWF	rs769080	-	-	-	0.00	0.00	0.00	0.00
**180**	NC_000012:5968688-5968688	C	intron_variant	VWF	rs2012829	-	-	-	0.00	0.00	0.00	0.00
**181**	NC_000012:5968656-5968656	C	intron_variant	VWF	rs12307110	-	-	-	0.00	0.00	0.00	0.00
**182**	NC_000012:5967775-5967775	G	intron_variant	VWF	rs216851	-	-	-	0.00	0.00	0.02	0.00
**183**	NC_000012:5965306-5965306	A	intron_variant	VWF	rs73049469	-	-	-	0.00	0.00	0.00	0.00
**184**	NC_000012:5964354-5964354	G	intron_variant	VWF	rs12369177	-	-	-	0.00	0.00	0.00	0.00
**185**	NC_000012:5964017-5964017	C	intron_variant	VWF	rs12307884	-	-	-	0.00	0.00	0.00	0.00
**186**	NC_000012:5964012-5964012	C	intron_variant	VWF	rs12307883	-	-	-	0.00	0.00	0.00	0.00
**187**	NC_000012:5962770-5962770	C	intron_variant	VWF	rs11063957	-	-	-	0.00	0.00	0.00	0.00
**188**	NC_000012:5962637-5962645	-	intron_variant	VWF	rs60444970	-	-	-	-	-	-	-
**189**	NC_000012:5961673-5961673	A	intron_variant	VWF	rs2363309	-	-	-	0.00	0.00	0.00	0.00
**190**	NC_000012:5958468-5958468	A	intron_variant	VWF	rs11063956	-	-	-	0.00	0.00	0.00	0.00
**191**	NC_000012:5958348-5958348	C	intron_variant	VWF	rs11063955	-	-	-	0.00	0.00	0.00	0.00
**192**	NC_000012:5957986-5957986	T	intron_variant	VWF	rs7964554	-	-	-	0.00	0.00	0.00	0.00
**193**	NC_000012:5957520-5957520	T	intron_variant	VWF	rs35926634	-	-	-	0.00	0.00	0.00	0.00
**194**	NC_000012:5957191-5957191	G	intron_variant	VWF	rs10849363	-	-	-	0.00	0.00	0.00	0.00
**195**	NC_000012:5955904-5955904	G	intron_variant	VWF	rs11063953	-	-	-	0.00	0.00	0.00	0.00

**196**	NC_000012:5955526-5955526	A	intron_variant	VWF	rs12819922	-	-	-	0.00	0.00	0.00	0.00
**197**	NC_000012:5954793-5954793	T	intron_variant	VWF	rs723189	-	-	-	0.00	0.00	0.00	0.00
**198**	NC_000012:5953976-5953976	T	intron_variant	VWF	rs12317523	-	-	-	0.00	0.00	0.00	0.00
**199**	NC_000012:5953817-5953817	A	intron_variant	VWF	rs7958883	-	-	-	0.00	0.00	0.00	0.00
**200**	NC_000012:5953723-5953723	G	intron_variant	VWF	rs12297442	-	-	-	0.00	0.00	0.00	0.00
**201**	NC_000012:5953712-5953712	A	intron_variant	VWF	rs3759321	-	-	-	0.02	0.00	0.00	0.00
			non_coding_transcript_variant									
**202**	NC_000012:5953687-5953687	T	intron_variant	VWF	rs3759320	-	-	-	0.00	0.00	0.00	0.00
			non_coding_transcript_variant									
**203**	NC_000012:5953213-5953213	G	intron_variant	VWF	rs12831864	-	-	-	0.00	0.00	0.00	0.00
			non_coding_transcript_variant									
**204**	NC_000012:5952781-5952781	C	intron_variant	VWF	rs7133777	-	-	-	0.00	0.00	0.01	0.00
			non_coding_transcript_variant									
**205**	NC_000012:5948745-5948745	G	upstream_gene_variant	ANO2	rs1990326	-	-	-	-	-	-	-
**206**	NC_000012:5947502-5947502	C	upstream_gene_variant	ANO2	rs11063951	-	-	-	-	-	-	-
**207**	NC_000012:5947326-5947326	C	upstream_gene_variant	ANO2	rs3809242	-	-	-	-	-	-	-
**208**	NC_000012:5947284-5947284	T	upstream_gene_variant	ANO2	rs3809241	-	-	-	-	-	-	-
**209**	NC_000012:5946627-5946630	-	upstream_gene_variant	ANO2	rs3842300	-	-	-	-	-	-	-
**210**	NC_000012:5945966-5945966	G	downstream_gene_variant	VWF	rs6489682	-	-	-	0.00	0.00	0.00	0.00
			Intron-Variant	ANO2								
**211**	NC_000012:5945874-5945874	T	downstream_gene_variant	VWF	rs6416322	-	-	-	0.00	0.00	0.00	0.00
			Intron Variant	ANO2								
**212**	NC_000012:5945582-5945582	C	downstream_gene_variant	VWF	rs6489681	-	-	-	0.00	0.00	0.01	0.00
			Intron Variant	ANO2								
**213**	NC_000012:5945229-5945229	G	downstream_gene_variant	VWF	rs6489680	-	-	-	0.00	0.00	0.00	0.00
			Intron Variant	ANO2								
**214**	NC_000012:5945034-5945034	A	downstream_gene_variant	VWF	rs117766816	-	-	-	0.00	0.00	0.00	0.00
			intron_variant	ANO2								0
**215**	NC_000012:5943702-5943702	T	intron_variant	ANO2	rs7314566	-	-	-	0.00	0.00	0.00	0.00

Table S1 presents a complete list of homozygous variants identified in VWF gene (*VWF*; chr12:5,948,877-6,124,770 [GRCh38/hg38]) after NGS and subsequent bioinformatics analysis by VEP, including variant annotation, variant location, the existing variant in public variant databases (SNPdb, SNPs database) and predicting its pathogenic effect. The impact of exonic variants was evaluated by SIFT and PolyPhen prediction tools, as well as splicing prediction tool SpliceAl. The intronic variants were only validated by Splice Al (Illumina artificial intelligence splicing prediction software). **SIFT** predicts whether an amino acid substitution affects protein function based on sequence homology and the physical properties of amino acids. PolyPhen is a tool that predicts the possible impact of an amino acid substitution on the structure and function of a human protein using straightforward physical and comparative considerations. For both SIFT and PolyPhen, VEP can report either a score between 0 and 1, a prediction in words, or both. SpliceAl calculations are including **DS_AG (**Delta score for acceptor gain), **DS_AL (**Delta score for acceptor loss), **DS_DG** (Delta score for donor gain), and **DS_DL** (Delta score for donor loss). The calculated scores range from 0 to 1 and can be interpreted as the probability of the variant being splice-altering. The suggested cutoffs are: 0.2 (high recall), 0.5 (recommended), and 0.8 (high precision)**.**

Table S2. Allele frequency data for homozygous variants located in von Willebrand factor (VWF) gene of the index patient, from genotyping project, the gnomAD

	Location	SNPdb ID	Allele	SYMBOL	AFR_AF	AMR_AF	EAS_AF	EUR_AF	SAS_AF
**1**	NC_000012:6121122-6121122	rs6489694	G	VWF	1	0.9957	1	0.9841	0.9847
**2**	NC_000012:6116076-6116076	rs4764537	G	VWF	1	0.9957	1	0.9841	0.9857
**3**	NC_000012:6107670-6107670	rs4485164	T	VWF	0.7791	0.5519	0.6349	0.7376	0.6892
**4**	NC_000012:6095736-6095737	rs5796209	-	VWF	0.2141	0.5259	0.7222	0.66	0.7362
**5**	NC_000012:6093442-6093442	rs2058223	A	VWF	0.4773	0.5793	0.7113	0.6968	0.7761
**6**	NC_000012:6093296-6093296	rs12317079	G	VWF	0.1929	0.5144	0.7113	0.6282	0.7219
**7**	NC_000012:6092634-6092634	rs142695490	A	VWF	-	-	-	-	-
**8**	NC_000012:6092632-6092632	rs148892733	A	VWF	-	-	-	-	-
**9**	NC_000012:6092630-6092630	rs143663161	A	VWF	-	-	-	-	-
**10**	NC_000012:6092628-6092628	rs144959320	A	VWF	-	-	-	-	-
**11**	NC_000012:6092626-6092626	rs150759772	A	VWF	-	-	-	-	-
**12**	NC_000012:6092624-6092624	rs144868844	A	VWF	-	-	-	-	-
**13**	NC_000012:6092622-6092622	rs145245519	A	VWF	-	-	-	-	-
**14**	NC_000012:6092620-6092620	rs147129737	A	VWF	-	-	-	-	-
**15**	NC_000012:6092519-6092519	rs3063460	G	VWF	-	-	-	-	-
**16**	NC_000012:6092515-6092515	rs66551998	TG	VWF	-	-	-	-	-
**17**	NC_000012:6092028-6092028	rs2239139	G	VWF	0.9395	0.6383	0.753	0.6998	0.7822
**18**	NC_000012:6091277-6091277	rs2239140	T	VWF	0.0356	0.4207	0.3026	0.495	0.3517
**19**	NC_000012:6089912-6089912	rs2239142	C	VWF	0.0363	0.428	0.2867	0.5	0.3609
**20**	NC_000012:6089774-6089774	rs1034933	G	VWF	0.9705	0.6326	0.7827	0.6988	0.7883
**21**	NC_000012:6089557-6089557	rs2109119	T	VWF	0.4236	0.5807	0.753	0.6968	0.7863
**22**	NC_000012:6089081-6089081	rs10849382	T	VWF	0.4682	0.5879	0.7897	0.6958	0.7883
**23**	NC_000012:6089075-6089075	rs10849381	T	VWF	0.4682	0.5879	0.7897	0.6958	0.7904
**24**	NC_000012:6089065-6089065	rs2238101	G	VWF	0.4168	0.5865	0.7609	0.6948	0.7863
**25**	NC_000012:6088302-6088302	rs2238102	C	VWF	0.9735	0.647	0.7867	0.7018	0.7914
**26**	NC_000012:6085489-6085489	rs2109118	C	VWF	0.0386	0.451	0.2857	0.498	0.3333
**27**	NC_000012:6085077-6085077	rs1860364	G	VWF	0.9599	0.6614	0.7946	0.7048	0.7658
**28**	NC_000012:6085017-6085017	rs2238103	A	VWF	0.7496	0.6066	0.7917	0.6362	0.6963
**29**	NC_000012:6084371-6084371	rs763580	G	VWF	0.9599	0.6484	0.7956	0.7008	0.7658
**30**	NC_000012:6083930-6083930	rs3819537	T	VWF	0.9599	0.6484	0.7956	0.6998	0.7658
**31**	NC_000012:6083720-6083720	rs3819538	G	VWF	0.9599	0.6484	0.7956	0.7008	0.7658
**32**	NC_000012:6081339-6081339	rs7133854	T	VWF	0.6067	0.5331	0.2897	0.5616	0.4039
**34**	NC_000012:6070224-6070224	rs137873960	T	VWF	0.0023	0.0029	0	0.0159	0
**35**	NC_000012:6066859-6066859	rs2238106	C	VWF	0.27	0.4323	0.6339	0.6163	0.6043
**36**	NC_000012:6063182-6063182	rs2239159	A	VWF	0.3964	0.245	0.4306	0.3837	0.5348
**37**	NC_000012:6062336-6062336	rs2191161	G	VWF	0.5234	0.5432	0.6647	0.7425	0.6902
**38**	NC_000012:6060567-6060567	rs4764482	C	VWF	0.1346	0.611	0.6915	0.5278	0.6074
**39**	NC_000012:6060277-6060277	rs980133	A	VWF	0.6921	0.7205	0.7222	0.6123	0.7249
**40**	NC_000012:6060032-6060032	rs980131	C	VWF	0.6921	0.7176	0.7242	0.6123	0.7249
**41**	NC_000012:6059861-6059861	rs980130	C	VWF	0.7738	0.7695	0.8879	0.6829	0.7955
**42**	NC_000012:6054787-6054787	rs36100099	A	VWF	0.3154	0.621	0.6845	0.493	0.6033
**43**	NC_000012:6053686-6053686	rs2283333	C	VWF	0.8964	0.8905	0.7679	0.8887	0.8783
**44**	NC_000012:6053588-6053588	rs2239160	G	VWF	0.8533	0.8948	0.7669	0.8887	0.8783
**45**	NC_000012:6053495-6053495	rs2239161	A	VWF	0.9599	0.902	0.7649	0.8887	0.8773

**46**	NC_000012:6051577-6051578	rs386759880	TG	VWF	-	-	-	-	-
**47**	NC_000012:6051526-6051526	rs4764531	A	VWF	0.3359	0.6542	0.6796	0.5338	0.6861
**48**	NC_000012:6051039-6051039	rs75336349	Ins.	VWF	0.3933	0.6657	0.6806	0.5517	0.681
**49**	NC_000012:6050980-6050980	rs11064010	C	VWF	0.6679	0.8602	0.7708	0.8767	0.863
**50**	NC_000012:6050879-6050879	rs10849379	T	VWF	0.382	0.6686	0.6825	0.5537	0.6851
**51**	NC_000012:6050371-6050371	rs216304	G	VWF	0.851	0.9107	0.7738	0.9085	0.91
**52**	NC_000012:6049879-6049879	rs216303	T	VWF	0.8525	0.9107	0.7738	0.9095	0.9018
**53**	NC_000012:6048856-6048856	rs216302	A	VWF	0.8517	0.9107	0.7758	0.9095	0.9029
**54**	NC_000012:6048822-6048822	rs216301	G	VWF	0.8517	0.9107	0.7758	0.9095	0.9029
**55**	NC_000012:6047469-6047469	rs216299	G	VWF	0.8343	0.9049	0.7758	0.9095	0.9029
**56**	NC_000012:6047311-6047311	rs216298	T	VWF	0.8343	0.9035	0.7758	0.9085	0.9018
**57**	NC_000012:6045504-6045504	rs216296	G	VWF	0.9614	0.9193	0.7778	0.9095	0.9029
**58**	NC_000012:6045040-6045040	rs216295	C	VWF	0.8101	0.8977	0.7778	0.9095	0.9029
**59**	NC_000012:6044584-6044584	rs216294	G	VWF	0.9614	0.9222	0.7778	0.9095	0.9029
**60**	NC_000012:6044493-6044493	rs216293	T	VWF	0.2171	0.67	0.6865	0.5417	0.684
**61**	NC_000012:6044098-6044098	rs11064008	G	VWF	0.0023	0.0937	0	0.1521	0.0266
**62**	NC_000012:6043810-6043810	rs216292	A	VWF	0.1725	0.6599	0.6865	0.5427	0.684
**63**	NC_000012:6043683-6043683	rs73034873	G	VWF	0.0015	0.0144	0	0.0199	0.001
**64**	NC_000012:6042707-6042707	rs216291	T	VWF	0.1823	0.6671	0.6855	0.5437	0.7055
**65**	NC_000012:6042431-6042431	rs216290	A	VWF	0.7511	0.8775	0.7768	0.8757	0.864
**66**	NC_000012:6041658-6041658	rs183356	A	VWF	0.8533	0.915	0.7768	0.9105	0.9018
**67**	NC_000012:6040118-6040118	rs216339	C	VWF	0.9735	0.9323	0.7917	0.9135	0.8885
**68**	NC_000012:6039160-6039160	rs216338	G	VWF	0.7194	0.7219	0.7877	0.6243	0.7587
**69**	NC_000012:6039143-6039143	rs216337	C	VWF	0.6694	0.7147	0.7877	0.6243	0.7546
**70**	NC_000012:6039064-6039064	rs216336	G	VWF	0.7171	0.7205	0.7877	0.6233	0.7587
**71**	NC_000012:6038839-6038839	rs216335	C	VWF	0.9667	0.9265	0.7907	0.9115	0.8824
**72**	NC_000012:6038445-6038445	rs216334	T	VWF	0.7723	0.745	0.7877	0.6521	0.772
**73**	NC_000012:6037953-6037953	rs216333	C	VWF	0.7731	0.745	0.7877	0.6541	0.7751
**74**	NC_000012:6037890-6037890	rs216332	A	VWF	0.7776	0.745	0.7867	0.6541	0.774
**75**	NC_000012:6037848-6037848	rs216331	T	VWF	0.7776	0.745	0.7867	0.6541	0.773
**76**	NC_000012:6037464-6037464	rs216330	C	VWF	0.646	0.7421	0.7867	0.6541	0.774
**77**	NC_000012:6037341-6037341	rs216329	C	VWF	0.8064	0.7507	0.7877	0.6541	0.774
**78**	NC_000012:6037317-6037317	rs216328	T	VWF	0.8064	0.7507	0.7877	0.6541	0.773
**79**	NC_000012:6036983-6036983	rs216327	C	VWF	0.8071	0.7507	0.7867	0.6541	0.774
**80**	NC_000012:6036782-6036782	rs216326	A	VWF	0.5628	0.6744	0.7143	0.5606	0.6155
**81**	NC_000012:6036363-6036363	rs216325	A	VWF	0.795	0.7507	0.7808	0.6521	0.774
**82**	NC_000012:6036059-6036059	rs216324	G	VWF	0.9448	0.9265	0.7887	0.9115	0.8824
**83**	NC_000012:6035943-6035943	rs216323	T	VWF	0.6354	0.7435	0.7867	0.6551	0.774
**84**	NC_000012:6035731-6035731	rs216322	T	VWF	0.6369	0.7435	0.7867	0.6541	0.7832
**85**	NC_000012:6035003-6035003	rs669667	G	VWF	0.9448	0.9265	0.7917	0.9145	0.8845
**86**	NC_000012:6034998-6034998	rs499648	C	VWF	0.9448	0.9265	0.7917	0.9145	0.8845
**87**	NC_000012:6034818-6034818	rs216321	C	VWF	0.9584	0.9265	0.7907	0.9135	0.8855
**88**	NC_000012:6034775-6034775	rs756662315	T	VWF	-	-	-	-	-
**89**	NC_000012:6034190-6034190	rs216320	C	VWF	0.9463	0.9265	0.7907	0.9135	0.8845
**90**	NC_000012:6032875-6032875	rs796891068	AC	VWF	0.7148	0.7219	0.754	0.6412	0.7546
**91**	NC_000012:6030095-6030095	rs216318	C	VWF	0.9667	0.9265	0.7956	0.9135	0.8865
**92**	NC_000012:6027748-6027748	rs216315	G	VWF	0.9743	0.928	0.7956	0.9125	0.8845

**93**	NC_000012:6027414-6027414	rs216314	C	VWF	0.9667	0.9265	0.7917	0.9135	0.8855
**94**	NC_000012:6019277-6019277	rs216311	C	VWF	0.9228	0.7752	0.746	0.6014	0.7863
**95**	NC_000012:6018777-6018777	rs216310	G	VWF	0.9221	0.7752	0.746	0.6014	0.7924
**96**	NC_000012:6017761-6017761	rs216309	T	VWF	0.326	0.6556	0.6349	0.502	0.6339
**97**	NC_000012:6015500-6015500	rs216308	C	VWF	0.9145	0.7464	0.75	0.5895	0.7924
**98**	NC_000012:6011912-6011912	rs216305	T	VWF	0.7005	0.6816	0.6419	0.498	0.6452
**99**	NC_000012:6009094-6009094	rs216814	T	VWF	0.5696	0.6441	0.7073	0.5129	0.6022
**100**	NC_000012:6008617-6008617	rs216813	G	VWF	0.6044	0.6542	0.7073	0.5179	0.6094
**101**	NC_000012:6005478-6005478	rs216810	C	VWF	0.646	0.6542	0.7183	0.5219	0.6043
**102**	NC_000012:6004278-6004278	rs216808	C	VWF	0.646	0.6542	0.7173	0.5209	0.6002
**103**	NC_000012:6003290-6003290	rs216807	A	VWF	0.6021	0.6527	0.7173	0.5199	0.5992
**104**	NC_000012:6002359-6002359	rs216806	G	VWF	0.6467	0.6542	0.7163	0.5219	0.6002
**105**	NC_000012:6002245-6002245	rs216804	A	VWF	0.646	0.6542	0.7163	0.5209	0.6002
**106**	NC_000012:6000796-6000796	rs188819154	T	VWF	0.0401	0.2478	0.3651	0.0616	0.138
**107**	NC_000012:6000790-6000792	rs149367153	-	VWF	0.2769	0.4611	0.7212	0.3678	0.5409
**108**	NC_000012:6000736-6000736	rs216803	C	VWF	0.6021	0.5965	0.7173	0.5159	0.6207
**109**	NC_000012:5999937-5999937	rs216802	A	VWF	0.5991	0.5965	0.7262	0.5159	0.6033
**110**	NC_000012:5999347-5999347	rs216801	A	VWF	0.2814	0.4683	0.7262	0.3708	0.5409
**111**	NC_000012:5998780-5998780	rs216800	T	VWF	0.2655	0.3674	0.4821	0.2555	0.409
**112**	NC_000012:5998451-5998451	rs7398722	C	VWF	0.997	1	1	1	1
**113**	NC_000012:5996852-5996852	rs216904	C	VWF	0.2806	0.4222	0.7262	0.3688	0.545
**114**	NC_000012:5996333-5996333	rs216903	C	VWF	0.6029	0.5504	0.7272	0.5159	0.6104
**115**	NC_000012:5996221-5996221	rs216902	A	VWF	0.2784	0.4179	0.7252	0.3648	0.5399
**116**	NC_000012:5995702-5995702	rs216901	A	VWF	0.4009	0.5259	0.7262	0.5109	0.6053
**117**	NC_000012:5994832-5994832	rs12099542	T	VWF	0.0408	0.2003	0.375	0.0606	0.1411
**118**	NC_000012:5993598-5993598	rs216898	A	VWF	0.6437	0.6542	0.7272	0.5119	0.6084
**119**	NC_000012:5993441-5993441	rs216897	C	VWF	0.4017	0.6311	0.7272	0.5129	0.6104
**120**	NC_000012:5991630-5991630	rs216896	T	VWF	0.4062	0.5259	0.7262	0.5129	0.6043
**121**	NC_000012:5991429-5991429	rs216895	T	VWF	0.4092	0.5245	0.7252	0.5139	0.6104
**122**	NC_000012:5991411-5991411	rs216894	G	VWF	0.41	0.5836	0.7252	0.5139	0.6115
**123**	NC_000012:5991319-5991319	rs216893	G	VWF	0.4319	0.5245	0.7252	0.5139	0.6094
**124**	NC_000012:5990909-5990909	rs4348960	A	VWF	-	-	-	-	-
**125**	NC_000012:5990862-5990862	rs216892	A	VWF	0.3253	0.5115	0.7232	0.5139	0.6125
**126**	NC_000012:5990683-5990683	rs216891	G	VWF	0.326	0.5187	0.7252	0.5159	0.6309
**127**	NC_000012:5990372-5990372	rs216890	C	VWF	0.4319	0.5245	0.7252	0.5139	0.6115
**128**	NC_000012:5990287-5990287	rs216889	C	VWF	0.6346	0.5461	0.7252	0.5149	0.6104
**129**	NC_000012:5989691-5989691	rs216888	G	VWF	0.4327	0.5245	0.7242	0.5139	0.6115
**130**	NC_000012:5989286-5989286	rs216887	C	VWF	0.4327	0.5245	0.7252	0.5149	0.6115
**131**	NC_000012:5988799-5988799	rs216886	T	VWF	0.4289	0.5317	0.7242	0.5119	0.6094
**132**	NC_000012:5988789-5988789	rs216885	C	VWF	0.3722	0.4741	0.5	0.3986	0.4888
**133**	NC_000012:5988662-5988662	rs216884	A	VWF	0.3086	0.4568	0.4881	0.3996	0.4775
**134**	NC_000012:5987650-5987650	rs216882	T	VWF	0.5983	0.879	0.8998	0.8817	0.7965
**135**	NC_000012:5987546-5987546	rs216881	G	VWF	0.6029	0.8847	0.8998	0.8887	0.7986
**136**	NC_000012:5987525-5987525	rs216880	A	VWF	0.6014	0.8847	0.8998	0.8867	0.7975
**137**	NC_000012:5987510-5987510	rs526881	A	VWF	0.6014	0.8862	0.8998	0.8877	0.7975
**138**	NC_000012:5987507-5987507	rs526861	G	VWF	0.5484	0.8256	0.6647	0.7704	0.6585
**139**	NC_000012:5987489-5987489	rs216879	T	VWF	0.6014	0.8862	0.8998	0.8867	0.7975

**140**	NC_000012:5987217-5987217	rs216878	C	VWF	0.6498	0.8862	0.8998	0.8817	0.7965
**141**	NC_000012:5987128-5987128	rs216877	C	VWF	0.6498	0.8862	0.8998	0.8817	0.7965
**142**	NC_000012:5987090-5987090	rs216876	A	VWF	0.6498	0.8862	0.8998	0.8817	0.7965
**143**	NC_000012:5986735-5986735	rs216875	G	VWF	0.6778	0.8862	0.8998	0.8817	0.7965
**144**	NC_000012:5986641-5986642	rs71450640	TT	VWF	-	-	-	-	-
**145**	NC_000012:5986591-5986591	rs216874	C	VWF	0.6793	0.8862	0.8998	0.8817	0.7955
**146**	NC_000012:5986108-5986108	rs216873	C	VWF	0.621	0.8343	0.6617	0.7714	0.6575
**147**	NC_000012:5985679-5985679	rs177702	A	VWF	0.6399	0.8372	0.6607	0.7694	0.6564
**148**	NC_000012:5984916-5984916	rs24177	A	VWF	0.1104	0.3602	0.4871	0.2545	0.408
**149**	NC_000012:5984374-5984374	rs216872	C	VWF	0.1112	0.3588	0.4861	0.2555	0.409
**150**	NC_000012:5983882-5983882	rs216870	A	VWF	0.4115	0.7305	0.6548	0.6272	0.59
**151**	NC_000012:5982531-5982531	rs190156617	C	VWF	0.0015	0.0101	0	0.0089	0
**152**	NC_000012:5981998-5981998	rs216868	A	VWF	0.1029	0.3559	0.4831	0.2535	0.4029
**153**	NC_000012:5981834-5981834	rs216867	G	VWF	0.708	0.8991	0.9028	0.8817	0.7924
**154**	NC_000012:5981137-5981137	rs216865	C	VWF	0.4228	0.732	0.6518	0.6272	0.5869
**155**	NC_000012:5978365-5978365	rs216859	C	VWF	0.6785	0.8991	0.8958	0.8847	0.7904
**156**	NC_000012:5977875-5977875	rs216858	A	VWF	0.2723	0.6988	0.6389	0.6034	0.5573
**157**	NC_000012:5977770-5977770	rs216857	T	VWF	0.4614	0.732	0.6528	0.6292	0.5869
**158**	NC_000012:5977099-5977099	rs216856	C	VWF	0.4228	0.7305	0.6528	0.6272	0.5869
**159**	NC_000012:5976347-5976347	rs216855	G	VWF	0.4841	0.7824	0.8968	0.7425	0.726
**160**	NC_000012:5975698-5975698	rs216854	C	VWF	0.5666	0.7435	0.6478	0.6312	0.5869
**161**	NC_000012:5975547-5975547	rs216853	A	VWF	0.475	0.7334	0.6478	0.6312	0.5859
**162**	NC_000012:5975157-5975157	rs4764521	G	VWF	0.9629	0.8674	0.9206	0.8519	0.774
**163**	NC_000012:5975025-5975025	rs216852	C	VWF	0.3048	0.7406	0.6508	0.6491	0.6145
**164**	NC_000012:5973633-5973633	rs12829271	G	VWF	0.8449	0.5836	0.2956	0.7097	0.5286
**165**	NC_000012:5973586-5973586	rs10849366	G	VWF	0.7912	0.5807	0.2946	0.7097	0.5286
**166**	NC_000012:5973392-5973392	rs2239138	T	VWF	0.798	0.5807	0.2946	0.6988	0.5266
**167**	NC_000012:5972947-5972947	rs917859	G	VWF	0.7973	0.5807	0.2946	0.6988	0.5256
**168**	NC_000012:5972772-5972772	rs917858	T	VWF	0.7489	0.5735	0.2946	0.6918	0.5256
**169**	NC_000012:5971429-5971429	rs11063965	A	VWF	0.7958	0.5793	0.2927	0.6998	0.5245
**170**	NC_000012:5971411-5971411	rs2238110	C	VWF	0.7973	0.5793	0.2927	0.6998	0.5245
**171**	NC_000012:5971368-5971368	rs10849365	C	VWF	0.7519	0.5764	0.2927	0.6998	0.5245
**172**	NC_000012:5971124-5971124	rs7301070	A	VWF	0.7474	0.5764	0.2927	0.6988	0.5215
**173**	NC_000012:5970158-5970158	rs60982029	T	VWF	0.1974	0.1311	0.0159	0.1352	0.1493
**174**	NC_000012:5969892-5969892	rs12300917	T	VWF	0.1899	0.1686	0.0109	0.2038	0.181
**175**	NC_000012:5969772-5969772	rs11063964	G	VWF	0.4811	0.2334	0.1319	0.2525	0.2526
**176**	NC_000012:5969450-5969450	rs2270239	G	VWF	0.9644	0.572	0.3085	0.674	0.5286
**177**	NC_000012:5969258-5969258	rs35335161	T	VWF	0.0151	0.0346	0.001	0.0517	0.001
**178**	NC_000012:5968959-5968959	rs4764478	A	VWF	0.261	0.1729	0.0625	0.2316	0.2485
**179**	NC_000012:5968741-5968741	rs769080	A	VWF	0.9251	0.572	0.244	0.659	0.5153
**180**	NC_000012:5968688-5968688	rs2012829	C	VWF	0.9614	0.5778	0.245	0.661	0.5174
**181**	NC_000012:5968656-5968656	rs12307110	C	VWF	0.3533	0.1427	0.0218	0.1402	0.1595
**182**	NC_000012:5967775-5967775	rs216851	G	VWF	1	1	1	0.996	0.998
**183**	NC_000012:5965306-5965306	rs73049469	A	VWF	0.1702	0.1225	0.0218	0.1083	0.1973
**184**	NC_000012:5964354-5964354	rs12369177	G	VWF	0.7837	0.5605	0.2282	0.668	0.5971
**185**	NC_000012:5964017-5964017	rs12307884	C	VWF	0.3502	0.219	0.0248	0.3052	0.2474
**186**	NC_000012:5964012-5964012	rs12307883	C	VWF	0.6263	0.4539	0.1766	0.5258	0.4519

**187**	NC_000012:5962770-5962770	rs11063957	C	VWF	0.9539	0.4712	0.2192	0.6223	0.684
**188**	NC_000012:5962637-5962645	rs60444970	-	VWF	0.9047	0.3573	0.0367	0.4374	0.5941
**189**	NC_000012:5961673-5961673	rs2363309	A	VWF	0.7943	0.3444	0.0357	0.4314	0.59
**190**	NC_000012:5958468-5958468	rs11063956	A	VWF	0.2352	0.1816	0.0149	0.2684	0.3487
**191**	NC_000012:5958348-5958348	rs11063955	C	VWF	0.6604	0.2781	0.0337	0.3807	0.4693
**192**	NC_000012:5957986-5957986	rs7964554	T	VWF	0.6536	0.2781	0.0347	0.3807	0.4693
**193**	NC_000012:5957520-5957520	rs35926634	T	VWF	0.177	0.1744	0.0149	0.2684	0.3497
**194**	NC_000012:5957191-5957191	rs10849363	G	VWF	0.9062	0.3573	0.0367	0.4384	0.5951
**195**	NC_000012:5955904-5955904	rs11063953	G	VWF	0.2504	0.1859	0.0149	0.2694	0.3507
**196**	NC_000012:5955526-5955526	rs12819922	A	VWF	0.2496	0.1859	0.0149	0.2684	0.3497
**197**	NC_000012:5954793-5954793	rs723189	T	VWF	0.9531	0.4712	0.2163	0.6233	0.684
**198**	NC_000012:5953976-5953976	rs12317523	T	VWF	0.4047	0.2017	0.0159	0.2684	0.3497
**199**	NC_000012:5953817-5953817	rs7958883	A	VWF	0.9508	0.4712	0.2173	0.6243	0.684
**200**	NC_000012:5953723-5953723	rs12297442	G	VWF	0.2731	0.1873	0.0149	0.2684	0.3507
**201**	NC_000012:5953712-5953712	rs3759321	A	VWF	0.5091	0.3718	0.2004	0.5109	0.5644
**202**	NC_000012:5953687-5953687	rs3759320	T	VWF	0.8185	0.4568	0.2173	0.6233	0.684
**203**	NC_000012:5953213-5953213	rs12831864	G	VWF	0.9531	0.4712	0.2173	0.6233	0.684
**204**	NC_000012:5952781-5952781	rs7133777	C	VWF	0.9531	0.4712	0.2183	0.6233	0.684
**205**	NC_000012:5948745-5948745	rs1990326	G	VWF ANO2	0.9531	0.4726	0.2173	0.6233	0.684
**206**	NC_000012:5947502-5947502	rs11063951	C	VWF ANO2	0.1717	0.1744	0.0149	0.2694	0.3497
**207**	NC_000012:5947326-5947326	rs3809242	C	VWF ANO2	0.7814	0.8689	0.9792	0.838	0.7607
**208**	NC_000012:5947284-5947284	rs3809241	T	VWF ANO2	0.7769	0.866	0.9792	0.839	0.7607
**209**	NC_000012:5946627-5946630	rs3842300	- -	VWF ANO2	0.1838	0.1758	0.0149	0.2694	0.3497
**210**	NC_000012:5945966-5945966	rs6489682	G	VWF ANO2	0.7209	0.8516	0.9792	0.832	0.7505
**211**	NC_000012:5945874-5945874	rs6416322	T	VWF ANO2	0.7201	0.8516	0.9792	0.832	0.7505
**212**	NC_000012:5945582-5945582	rs6489681	C	VWF ANO2	0.8616	0.9885	1	1	1
**213**	NC_000012:5945229-5945229	rs6489680	G	VWF ANO2	0.59	0.9063	0.8383	0.9493	0.8742
**214**	NC_000012:5945034-5945034	rs117766816	A	VWF ANO2	0.0023	0.0144	0	0.0328	0.001
**215**	NC_000012:5943702-5943702	rs7314566	T	ANO2	0.1868	0.2767	0.1944	0.4592	0.4264

Ensemble Variant Effect Predictor (VEP) report allele frequency (AF) data for existing variants of the von Willebrand factor (gene) of index patient from genotyping project, the **gnomAD**. AFR (African), AMR (American), EUR (European), EAS (East Asian), SAS (South Asian)
